# Automating multistep flow synthesis: approach and challenges in integrating chemistry, machines and logic

**DOI:** 10.3762/bjoc.13.97

**Published:** 2017-05-19

**Authors:** Chinmay A Shukla, Amol A Kulkarni

**Affiliations:** 1 Academy of Scientific and Innovative Research (AcSIR), CSIR-National Chemical Laboratory (NCL) Campus, Pune 411008, India; 2 Chem. Eng. & Proc. Dev. Div., CSIR-National Chemical Laboratory, Dr. Homi Bhaba Road, Pashan, Pune 411008, India

**Keywords:** automation, control strategy, flow chemistry, in-line monitoring, multistep synthesis optimization

## Abstract

The implementation of automation in the multistep flow synthesis is essential for transforming laboratory-scale chemistry into a reliable industrial process. In this review, we briefly introduce the role of automation based on its application in synthesis viz. auto sampling and inline monitoring, optimization and process control. Subsequently, we have critically reviewed a few multistep flow synthesis and suggested a possible control strategy to be implemented so that it helps to reliably transfer the laboratory-scale synthesis strategy to a pilot scale at its optimum conditions. Due to the vast literature in multistep synthesis, we have classified the literature and have identified the case studies based on few criteria viz. type of reaction, heating methods, processes involving in-line separation units, telescopic synthesis, processes involving in-line quenching and process with the smallest time scale of operation. This classification will cover the broader range in the multistep synthesis literature.

## Introduction

### Multistep flow synthesis

In the recent time the concept of flow chemistry has become an important milestone in organic and materials synthesis. It has also been proven to be successful for a large number of reactions and the natural evolution of flow synthesis was to extend for its applicability to complex chemistries and large molecules [1–4]. In general, the complexity of synthesis depends upon the method and/or a number of steps and/or specific functional activity, etc. Most of the useful synthetic organic compounds involve a series of chemical transformations of very different nature, which can be termed simply as ‘multistep synthesis’. The final products can have applications in fine chemicals, agrochemicals, and pharmaceuticals. Multistep syntheses enable the synthesis of complex molecules, which otherwise would be practically impossible if performed in a single step. In multistep flow synthesis, the general approach is to mix the reagents in a suitable micromixer followed by a flow reactor (usually depicted in the form of a helical coil or a packed column), which is maintained at a desired temperature or a given temperature profile. The outlet stream of the reactor is subsequently mixed with the new reagent and allowed to react for further transformation and so on [5–6]. The multistep synthesis may or may not involve inline separation units and also in-line analytical tools to monitor the process. The multistep synthesis where the intermediate separation or work-up is not required is conventionally termed as ‘one pot’ synthesis, which is also called ‘telescopic’ synthesis when carried out in a continuous mode. A separation or purification step is required if (i) there is a need to isolate the necessary phase or isomer or (ii) to switch to a new solvent due to chemical compatibility or (iii) due to an unviable boiling point or (iv) for the cases where the side-product/byproduct can significantly affect the yield of the subsequent reaction step.

Reactions involved in a multistep synthesis can be classified in many ways. The general approach for classification of reactions is based on the activation methods like radical reaction, electrophilic reaction, electrochemical reaction, photochemical reaction, microwave, etc. On the other hand, it is also possible and in many times necessary to classify the reactions on the basis of the number of phases (gas G, liquid L, and solid S) involved in the reaction (viz. single-phase or homogeneous reaction and multiphase reactions). In single-phase homogeneous reactions, the reactants and products are soluble in the solvent or the reaction medium. Multiphase reactions involve two or more immiscible phases like G-L [7], L-L [8], G-L-L, L-S [9] and G-L-S [4] reactions. Sometimes for such reactions, phase-transfer catalysts are involved in enhancing the mass transfer rates or even one of the products needs to be isolated continuously to shift the equilibrium.

Recently excellent literature has been published on the synthesis of high-value compounds using a multistep synthesis approach [3–4,7,9–24]. Several integrated protocols have been developed for generating a library of compounds [12,25–27]. In-line separators like scavenging columns [22,25–28], liquid–liquid extractors (based on gravity or membrane) [3,23,29], distillation [30], etc. also facilitate continuous separation and significantly reduce the time for process development. Recent reviews on multistep synthesis clearly highlight the potential application of multistep syntheses in fine and pharmaceutical industries [5,31–32]. However, while continuous-flow synthesis helps to reduce the reaction time scales significantly, complex work-up and offline analysis are some of the bottlenecks of easy implementation of multistep flow synthesis. It also brings the need for automation. Automation in chemical synthesis is not new for the chemical industry. However, for the multistep flow synthesis of high-value molecules where each reaction step demands a very different set of optimal parameters to maximize the yield for that step, automating the synthesis approach will help in integrating the decision making, design of experiments and actual synthesis [33]. This will also help to identify the limitations of combining (and not integrating) reactions with separations/work-up. Usually, automation is not as straightforward as it gets depicted from the existing literature on flow synthesis. Automation involves the development of protocols for analysis of a situation (based on the input information in terms of the desired conversion, selectivity, impurity profiling), real-time integration with the process analytical tools and decision-making protocols for identifying the next set of conditions needed to move in the direction that leads to optimal performance (objective function). While these protocols can be implemented using a suitable software, necessarily embedded hardware with excellent accuracy that corroborates with the chemistry is also needed. It is evident that automation and machine based logical decision making will be the next logical evolution of flow synthesis, which would help in speeding up the optimization, process development and actual translation of chemistry to products.

This implies that integration of various core and peripheral domains from the relevant sciences and engineering is absolutely essential and unavoidable to automate on-demand and end-to-end synthesis of important molecules [34]. Interdisciplinary research with long-term sustainability objectives and scientific interactions with industries can only help to find useful solutions. This implies that the compartmental approach followed by the synthesis community (which is necessary only while conceiving a new creative chemistry) needs to be changed at a certain stage by looking at their creative invention as a process rather than remaining limited to lab-scale methods to be the first-to-demonstrate. First-to-demonstrate a complex synthesis should immediately or even right from the beginning should allow the approach to be looked through a process angle, which will help the creativity of synthesis to blossom into a process. This review paper uses multistep flow synthesis and automation as two different yet largely interdependent domains to show how an automated platform can be built to deliver more from synthesis, viz. in terms of data, consistency, and reproducibility.

## Review

### Automation in flow synthesis

Automation in target specific as well as routine chemical synthesis will be among the most likely happening things in the time to come [35–36]. The role of automation in the flow synthesis can be categorized into three levels. Each level or component of automation has a very different objective, complexity, and relevance to synthesis.

**I) Auto-sampling and analysis:** In such cases, there is no control structure or Design of Experiment (DoE). Here automation is responsible for in-line monitoring of reactants, intermediates or products at the outlet of the reactors or separators. It may also involve in-line measurement of other process parameters (often not shown in the literature) like temperature, pressure, pH, level, etc. [33]. Such automation is useful for screening a large library of potential drug candidates. For example, Guetzoyan et al. have demonstrated the use of automation for synthesizing imidazo[1,2-*a*]pyridines, potential GABA_A_ agonists [12].

**Figure 1:**
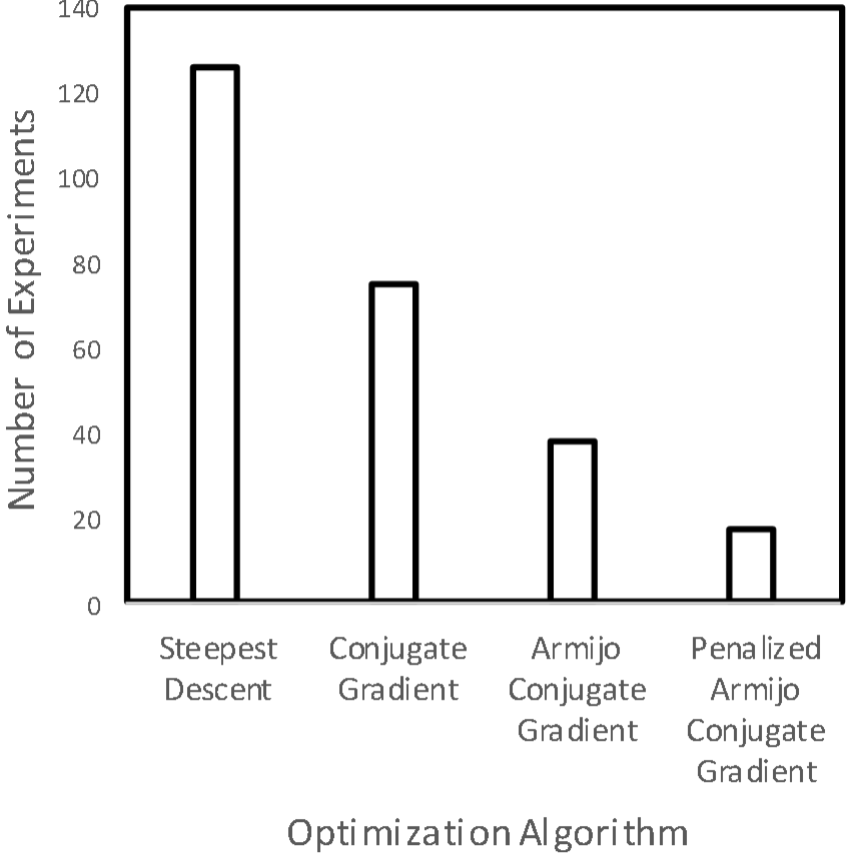
A number of experiments for various optimization algorithms [46].

**II) Optimization:** For a set synthesis protocol usually an optimization of conditions to maximize the yield of the desired product brings the need of repetitive work, which can be transformed to an automated synthesis platform (viz. vapourtec, H-cube, etc.). It is always possible to develop customized automation platforms that suit for specific synthesis and building such avenues using well-established programming tools like Lab View is always beneficial. In such cases, the DoE or optimization algorithm is coupled with automation protocols to find the optimal conditions. In-line analytical techniques can be coupled with a computer which manipulates the process conditions like temperature, flow rate, pressure, pH, etc., to achieve the desired objective (in most cases yield of the desired reaction) [37–40]. The control structure is not present in such cases as it does not have a real-time feedback system. An excellent review by Fabry et al. [41] on self-optimizing reactor systems is a useful resource to visualize the evolution of flow synthesis. However, as of now the self-optimizing reactor systems are limited to single-step syntheses and will need complex algorithms to evolve them to multistep syntheses. Although it is claimed that self-optimizing systems will lead to time and cost savings, selection of appropriate optimization algorithm and the algorithm development remains critical. Moore and Jensen have studied the optimization of a Paal–Knorr synthesis using various optimization algorithms [42]. Figure 1 shows the number of experiments for various optimization algorithms at an optimal temperature of 130 °C. The authors have clearly demonstrated that selecting the appropriate optimization algorithm is essential for minimizing the number of experiments thus saving time and resources. This analysis is extremely important when the reactants and reagents are extremely expensive or have a very small active life. However, if the number of experiments is going beyond 20–25, it would be advisable to generate kinetic data instead and use the appropriate chemical reaction engineering model to optimize the process [43]. Chemical reaction engineering models give more insight by allowing estimating the concentration and temperature profiles inside the reactor. Whenever it is not possible to insert temperature sensors along the flow reactor/microreactor due to its compact dimensions (although miniaturized sensors are available almost everywhere), the temperature should be monitored at the inlet (preferably at mixing points) and outlet of the reactor [44]. In such cases, reaction engineering models are useful for the prediction of the temperature profile inside the reactor and to investigate if any hot spot is occurring inside the reactor. Reizman and Jensen demonstrated the use of the automated platform for estimating kinetics parameters of a series-parallel substitution reaction [38]. Reizman et al. have studied Suzuki–Miyaura cross-coupling optimization using a DoE-based algorithm and feedback system [45]. The authors studied both continuous and discrete variables for optimization. Recently Fitzpatrick and Ley have demonstrated the use of automation for integrating batch and flow reactors on a single platform [46]. Their process also involved extraction and distillation operations.

**III) Automation for control:** The third and the most important purpose of automation is to control the process variables like temperature, pressure, and flow rate at the given set point so that it helps indirectly to control the reaction rates and pH of the reaction mixture. The objective of controlling certain parameters within a range usually needs accuracy in measurements as well as in terms of response time. In such cases, there is no DoE but the objective is to maintain a steady state process at optimal conditions through an appropriate control strategy. In general, automated control strategies are commonly implemented for bench scale, pilot scale, and commercial scale manufacturing of chemicals, and not at laboratory scale. However, this feature becomes important for multistep synthesis as for taking into account any feedback effect in the entire synthesis protocol, minor variation in the operating conditions at any stage can trigger a forward or backward effect leading to change in the reactor performance. Recently a few such works on the use of control systems for multistep synthesis of APIs and drugs are reported in the literature [3,8,19,24].

With this objective of implementing automation in synthesis, in the next section, we touch upon the automation at different scales and then focus on the possible ways in which multistep flow synthesis needs to be carried out to enhance the productivity and reliability of a synthesis protocol.

**Automation in lab-scale environment:** Automation can significantly improve the productivity of lab scale experiments and also aid speeding up the synthesis of a library of compounds and drug discovery process [47]. The chemical library has to go through high-throughput screening (about 100,000 compounds/day approximately) using robots [48]. In multistep synthesis off-line analysis actually becomes a bottleneck as it does not allow the real-time changes to be imposed at the inlet conditions for minor variations in the product quality. Automated in-line analysis has (to some extent) addressed this issue provided the response time from the systems is shorter than the time scales that control the reaction. Reliability and reproducibility of an experiment also improve significantly under automated environment [49]. With a significant portion of literature, falling under the non-reproducibility crisis, automation will make chemistry and methods as reliable as it was over several decades ago [50]. By using automation for measuring and reporting the scientific data, one can increase the value of the published work, patents, etc., by many folds. Automation is a powerful tool if used correctly, but can also be expensive if not utilized correctly [13].

**Automation in chemical plants:** The role of automation in chemical industries is to enhance the product quality, reduce the dependence on the availability of human being, improve process safety, efficiently utilize the plant resources and minimize the emissions [51]. Process automation is in great demand in various industrial sectors like chemical industry, power generation industry and petroleum industry. In the recent years, the pharmaceutical industries have been experiencing growing demands for process automation services like hardware and standard software. Stephanopoulos has reviewed the process control approach in chemical plants in detail [52]. The process control system should be designed to achieve the control objectives which are generally defined by the process or chemical engineers. The control objectives include both normal and special purpose operations. Normal operation during synthesis is controlling the process/reaction at optimum conditions. Special purpose operations may include start-up (viz. starting a continuous stirred tank reactor, adding a highly reactive reagent to the reactor, etc.), shut-down (viz. stopping the reaction, giving rapid cooling to the reaction mixture, etc.), change-over (viz. switching from reactant to solvent, changing or recovering the catalyst activity, etc.), override and emergency situations (viz. forcefully quenching the reaction). There should also be a sequence of operation procedures which can take the process from one state of operating conditions to another state of operation. Dynamic simulations can be a useful tool to study the special purpose operations (viz. start-up, shut-down, etc.) and also the forward and backward effects due to operating condition variations in the multistep synthesis. Understanding the forward and backward effects in multistep synthesis is essential for successful translation of chemistry into an industrial process.

Accurate measurements of process variables are the most critical part of the process control. The process variables that are often measured are temperature, pressure, flow, liquid level, density, composition, pH and viscosity. Details of different sensors or transmitters for measuring these variables can be found in standard process control and instrumentation textbooks [53–56]. Before automating any process it is necessary to understand that the simplest control system that will do the desired job is the best one and one must understand the process thoroughly before controlling it [57].

The fundamental step for designing a control system is to identify the controlled variable. A controlled variable can be the outlet temperature of a heat exchanger or a reactor, the outlet composition of the reactor, the system pressure, the liquid level of a tank or a crystallizer, the pH, etc. Selection of controlled variables is done by engineering judgment based on process understanding. The next step is to identify manipulating variables and formulate a control loop [52]. Generally, there are many options available in manipulation variables which make developing control loops challenging. As a thumb rule, flow rates are generally avoided as a manipulating variable when the flow rate is high or temperature is very high or the process stream is in slurry form (suspension of solids in liquids) or contains dosing of solids or has corrosive materials. Pressure is also generally avoided as a manipulating variable when the liquid is volatile or the process stream is a two-phase mixture. Recently, Movsisyan et al. have reviewed the application of flow reactors for hazardous reactions [1]. Although the flow reactors allow such reactions to be carried in a safer manner controlling such reactors at production scale could be challenging.

Recently, automation is also used for Hazard and Operability (HAZOP) analysis in chemical industries [58–59]. In HAZOP analysis, the aim is to systematically identify all the possible abnormal process deviations, its causes and its adverse effects in the chemical plant. HAZOP analysis is generally time-consuming and labour-intensive.

The recent advances in multistep synthesis have shown promising outcomes at lab scale especially for the synthesis of high-value drugs [4,7,11,17,21,23,60]. However, converting these chemistries into industrial processes is still challenging. While several industries provide solutions for scale-up of lab processes [61–67] usually their hands-on experience to decide the control strategy while automating a flow chemistry always offers better solutions than what one expects theoretically. Before analysing these complex syntheses, it is worth appreciating that a few successful demonstrations of an end-to-end manufacturing process for high-value drug compounds are already reported in the literature [3,8,19,24]. In this review, we have critically analysed a few multistep syntheses of high-value drugs using an approach that involves various unit operations like filtration, evaporation, membrane separation, liquid–liquid extraction, etc. We have also suggested some guidelines for these multistep syntheses for transforming these lab scale chemistries to automated pilot scale processes. At pilot or production scale, automation is largely employed for controlling and maintaining the process at a steady state. Here we have suggested some simple control strategies which can be adapted even for scale-up. With each process having different chemistries, unit operations and operating conditions, the operating protocol and control strategy may change every time making it a challenging task.

### Approach and selection criteria

The case studies discussed in the later section includes control strategies for various types of reactions, viz. homogeneous reactions, gas–liquid reactions, gas–liquid–solid reactions and also for various unit operations including heat exchangers, evaporators, membrane extractors, etc. However, similar control strategies can also be employed for other chemistries/processes which are not included in the present case studies. Each case study is transformed in the form of a Piping and Instrument Diagram (P&ID) that makes a process engineer understand the flow of processes and associated measurement instruments. The P&ID includes engineering details of equipment, instruments, piping, valves, fittings and their arrangements. It may also include identification numbers for the equipment, pipelines, pumps, and other auxiliary equipment. The most important feature of P&ID is that it provides a graphical representation of the control structure of the process [68–70]. In industry, P&ID is always made before actually implementing process automation and control for any process. More importantly, P&ID is also used for doing and evaluating HAZOP options which are critical to the implementation of a process.

### Case studies

Till date, there are more than 80 excellent publications that use continuous-flow synthesis of high-value molecules where the synthesis involves two and more stages. While choosing the representative case studies we have applied certain classification criteria such that it would cover the broader area in a multistep synthesis. A detailed analysis of more than 80 multistep flow syntheses reported in the literature will be presented separately. The first classification was based on a number of phases involved in the reaction, viz. gas–liquid reaction (viz. amitriptyline synthesis [7]), gas–liquid–solid reactions ((±)-oxomaritidine synthesis [9]) and liquid–solid reactions (viz. (*S*)-rolipram synthesis [4]). The process was also selected on the basis of the heating technique used, viz induction heating (olanzapine synthesis [10]), and conventional heating in a constant temperature bath/circulator. The cinnarizine/buclizine derivative process was selected as it involved in-line quenching [23]. Most of the above processes involve separation units and hence we decided to select telescopic synthesis of tamoxifen and rufinamide as representative case studies [11,14] that do not involve any phase separation. Finally, we selected an ibuprofen synthesis protocol as it had an overall residence time significantly less than the conventional approach [60].

#### Symbols for block diagram and P&ID

**Figure 2:**
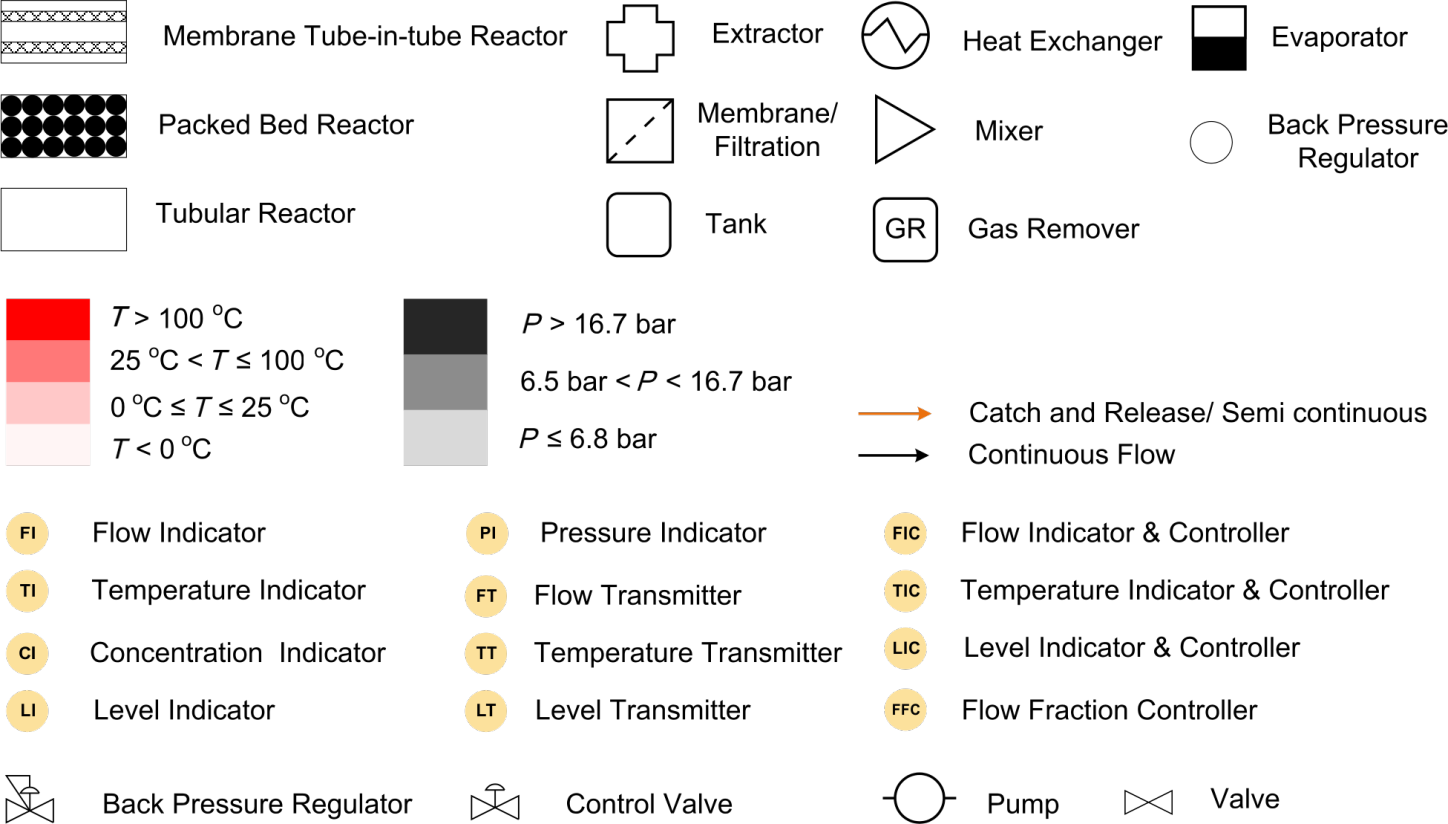
Symbols used for block and P&ID diagrams.

Translating any chemistry to process at industrial scale would require the involvement of engineers from various fields viz. chemical, instrumentation, mechanical and electrical. In such cases, it is desired to describe the process in terms of standard symbols (rather than combined chemical structures and diagrams which are most often used in the literature) which can be understood by process chemists as well as engineers from other disciplines. Figure 2 shows the list of symbols used in the current review. For each case, initially we have described the process chemistry and transformations followed by the approach that needs to be followed to make it a useful method that gives important data that can help it transform into an automated process.

#### Case study 1: Multistep flow synthesis of olanzapine/zyprexa (induction heating)

**Scheme 1:**
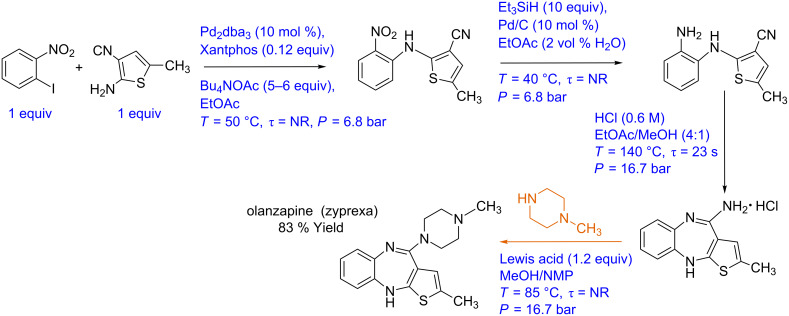
Multistep synthesis of olanzapine (Hartwig et al. [10])

**Figure 3:**
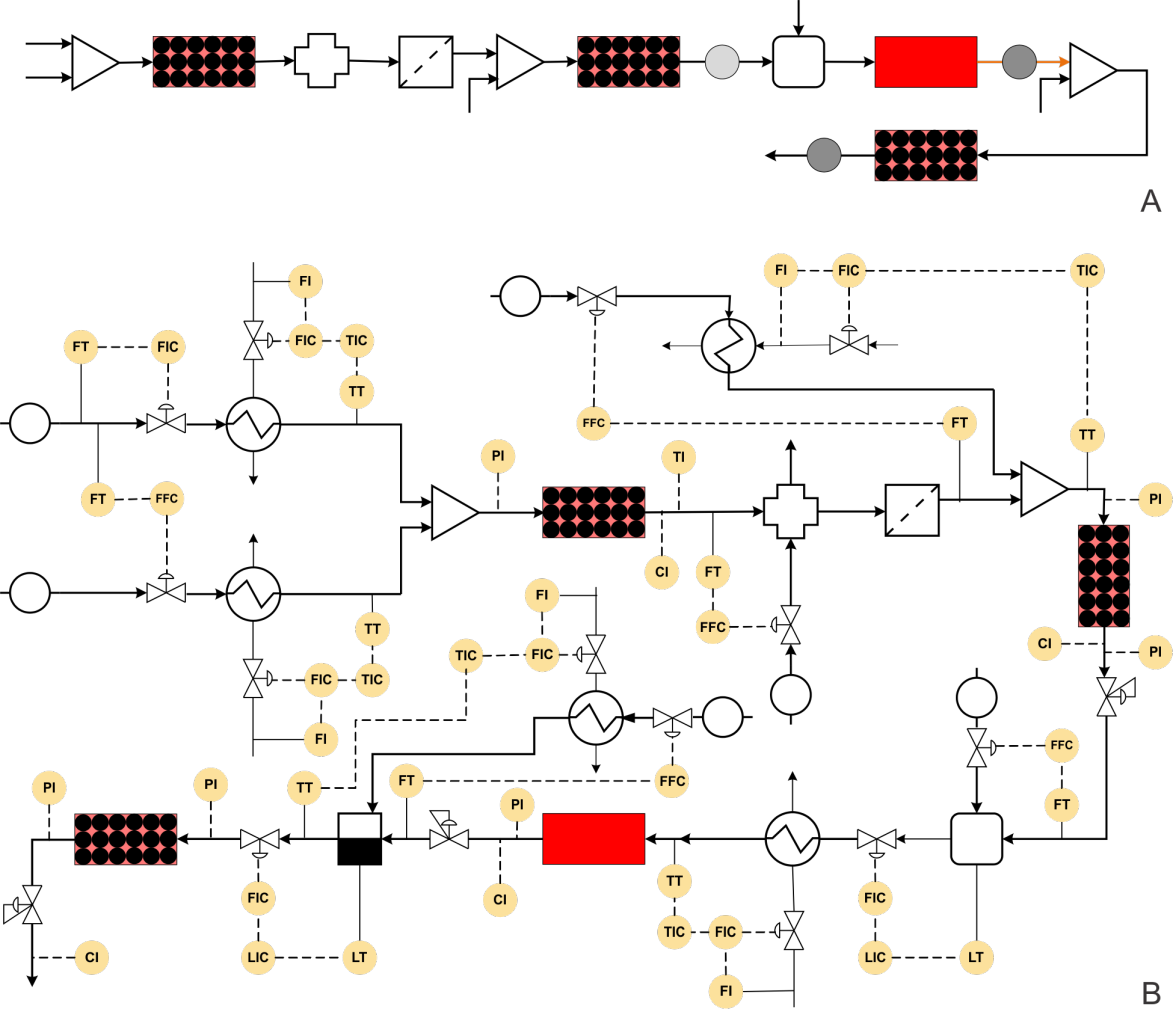
(A) Block diagram representation of the process shown in Scheme 1, (B) piping and instrumentation diagram of Scheme 1.

Kirsching and co-workers have reported the continuous multistep synthesis of olanzapine (Zyprexa), a drug used for the treatment of bipolar disorder and schizophrenia [10]. The process involves four reaction steps, one inline extraction, and a filtration step. The reaction is shown in Scheme 1. Initially, a Buchwald–Hartwig reaction is carried out between aryl iodide and aminothiazole. Pd_2_dba_3_ was used as a catalyst and Xantphos as a ligand in an ethyl acetate medium. These reagents are passed through a PEEK reactor filled with 0.8 mm steel beads and were heated at 50 °C by inductive heating (15 kHz). The reaction is subsequently quenched using distilled water and extracted in-line and passed through a silica cartridge to remove the Pd catalyst. The nitroaromatic derivative is mixed with triethylsilane and the mixture is passed through a fixed bed reactor with Pd/C catalyst at 40 °C to reduce the nitro group to an amino group. The yield is reported to be quantitative, and the catalyst activity is reported to be lasted for over 250 h. The reaction mixture is further mixed with HCl in MeOH/AcOEt and subjected to acid-catalyzed cyclization in a 0.3 mL coiled reactor at 140 °C (inductive heating, 800 kHz). This resulted in 88% overall yield. The isolated product is then mixed with piperazine and passed through the PEEK reactor (3 mL) containing MAGSILICA (inducting material) and silica-supported Ti(OiPr)_4_ (Lewis acid). The final substitution is reported at 85 °C (25 kHz) that gives olanzapine in 83% yield. These individual synthesis steps can be depicted in the form of a block diagram (Figure 3A). Since each synthesis step is carried out at a very different set of conditions, automation of such a synthesis scheme would need a lot of data from each step including the effects of minor variations in individual parameters at each step. Such an objective would need the intervention of several engineering inputs, which will help to transform this synthesis to a process.

Figure 3B shows the P&ID for the olanzapine manufacturing process. The flow rate of aryl iodide is fixed at a desired set point value using a control valve, which also helps to stop the pump in the case that the reaction temperature or pressure increase beyond a certain set-point over the subsequent reaction steps. The aminothiazole flow rate needs to be controlled using a ratio controller to maintain the molar ratio between aryl iodide and aminothiazole.

Both of these streams can be preheated using a heat exchanger with a feedback controller. The mixed stream can then be passed through the packed reactor with the inductive heating system. The induction heating system should actually be coupled with a suitable cooling system to avoid possible uneven spatial heating effects. Alternatively, unless otherwise needed, the conventional heating system can also be used as it will be more flexible in terms of operation and control. The outlet concentration of the intermediate can be monitored inline using a suitable analytical technique and the reactor conditions should be controlled by manipulating the reactor temperature. The reaction mixture can be quenched by distilled water and extracted in a column, where again the distilled water flow rate can be controlled using a ratio controller. Filtration can remove the Pd catalyst in a continuous manner. This process stream can be further mixed with preheated triethylsilane at appropriate molar ratio using a ratio controller.

This mixed stream can be passed through a packed bed reactor containing a Pd/C catalyst and maintained at 40 °C using a heating jacket. The reactor outlet concentration can be monitored inline and controlled by manipulating the jacket fluid flow rate of the reactor. The back pressure regulator can be used to maintain the desired pressure. Further, the HCl stream can be mixed with the process stream in a tank at appropriate molar ratio using a ratio controller. The outlet flow rate of the tank can be controlled by maintaining the liquid level inside the tank. The camera based level control systems demonstrated by Ley and co-workers can be a quick option [71]. The solution can be preheated to 140 °C using a heat exchanger and passed through a jacketed reactor. The outlet concentration of the reactor can be measured inline and controlled by manipulating the jacket fluid flow rate. This will help to measure the heat duty for the specific reaction, which can be used for estimating the enthalpy change in the specific reaction. This data is immensely useful for scale-up of this approach. A back pressure regulator (BPR) can be used to maintain the desired set pressure on the entire system. The process stream can be mixed with piperazine (in MeOH/NMP) and subjected to evaporation to remove ethyl acetate solvent. The outlet flow rate of the evaporator can be controlled by maintaining the liquid level inside the evaporator. The process stream can further be passed through a packed bed reactor containing silica-supported Ti(OiPr)_4_ maintained at 85 °C using a reactor jacket to obtain the olanzapine drug.

While the above mentioned automated protocol can be implemented for the synthesis in Scheme 1, it is not easy to implement a few aspects that are routinely used in the reported multistep synthesis. Here we bring out such challenges and suggest a few alternatives that will help an engineer to transform the protocol to synthesis. To begin with, the merit of this scheme comes from an efficient way of implementing inductive heating. Though inductive heating offers rapid heating, its applicability for large-scale synthesis is yet unreported and may not be economical. For exothermic reactions, the heating unit should also be capable of cooling in case of some undesired events like a runaway reaction or an emergency shutdown. Most of the induction heating systems cool at a slower rate due to natural convection and to some extent radiation. However, an additional set-up (for cooling the induction coil) is required for this which will add cost. Moreover, air needs to be very clean and possibly moisture free as it can corrode the induction coil. With this complex setup, it will be difficult to control the actual set point temperature inside the reactor. If there is a significant temperature difference between two consecutive reactors, then one can preheat (or cool) the process fluid to the desired temperature using conventional heating techniques and then pass it through the reactor for a better performance.

#### Case study 2: Multistep flow synthesis for tamoxifen (telescope synthesis)

**Scheme 2:**
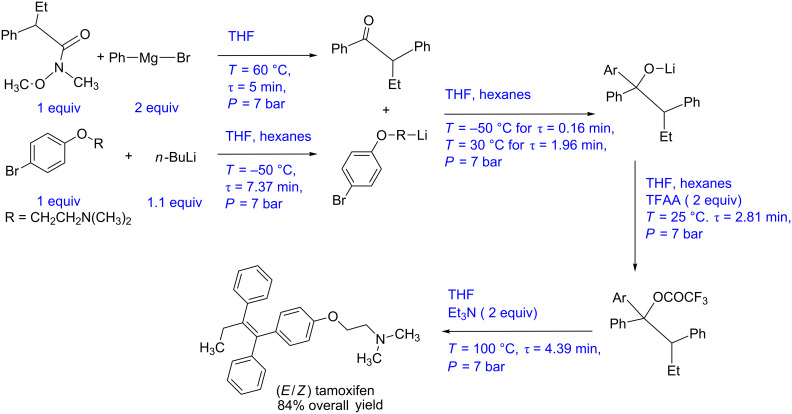
Multistep flow synthesis for tamoxifen (Murray et al. [11]).

**Figure 4:**
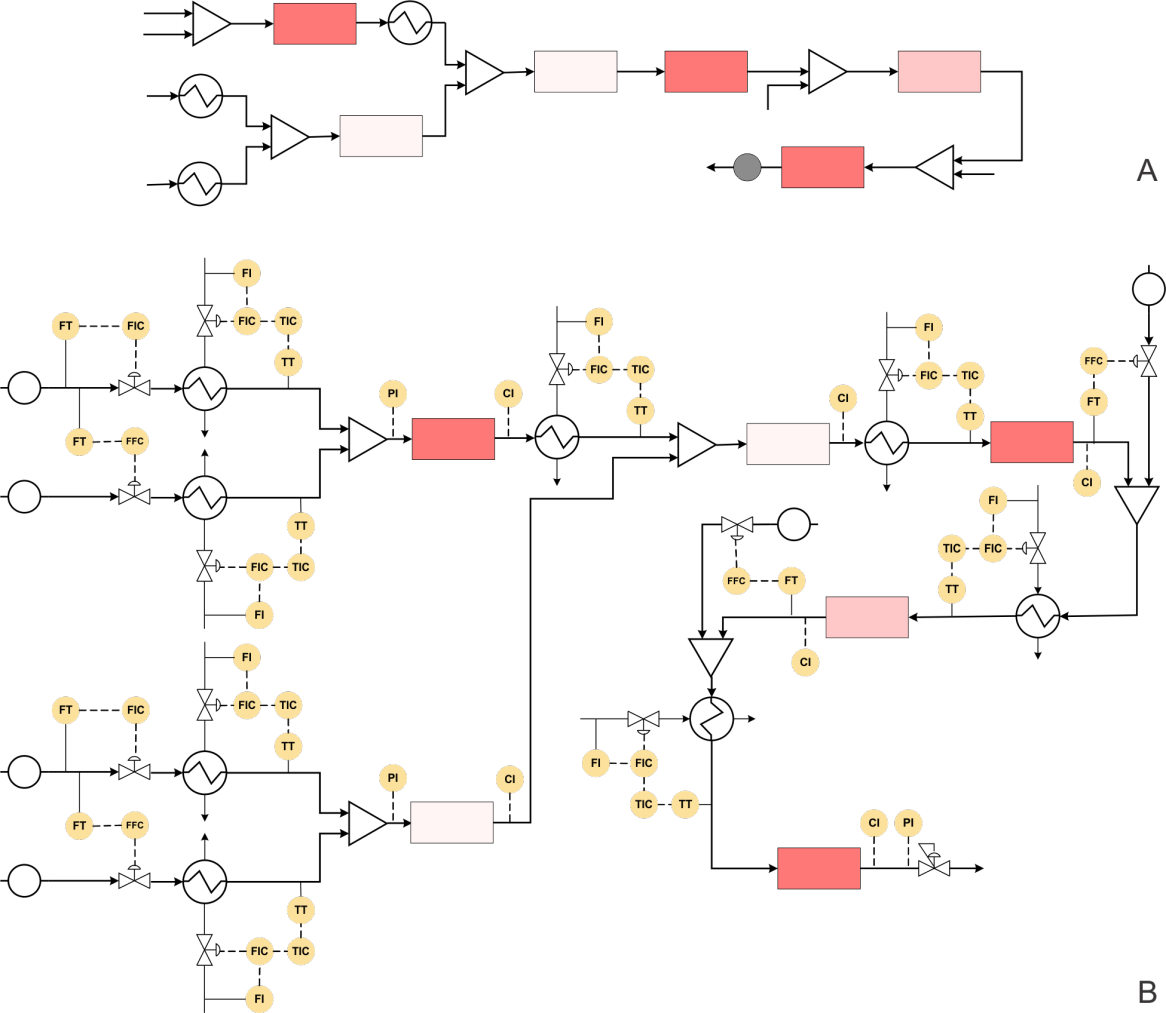
(A) Block diagram representation of the process shown in Scheme 2, (B) piping and instrumentation diagram of Scheme 2.

Steven Ley and co-workers have reported a continuous multistep synthesis protocol for tamoxifen, a drug used for treating breast cancer [11]. The synthesis protocol is shown in Scheme 2. A Weinreb amide (1 equiv) and PhMgBr (2 equiv, Grignard’s reagent) are reacted in a 10 mL PFA coil at 60 °C for 5 min residence time. The reaction is quenched using aq HCl and the ketone is isolated with 97% yield. The aryl lithium compound is produced simultaneously by reacting aryl bromide (1 equiv) with *n*-BuLi (1.1 equiv) at −50 °C in 10 mL PFA reactor with 7 min residence time. This lithium compound reacts with the ketone intermediate at 30 °C for 2 min in 5 mL PFA reactor coil. The intermediate lithium alkoxide is further reacted with trifluoroacetic anhydride (2 equiv) in a 10 mL PFA reactor at 25 °C for a residence time of 3 min to get the trifluoroacetate, which further reacts with triethylamine in a PFA coil (10 mL) at 100 °C for 5 min to give the final drug molecule. By using Vapourtec V-3 peristaltic pumps, the authors are able to achieve a constant fluid flow rate. Maintaining the mole ratio between ketone (obtained by Grignard’s addition) and lithiated compound (obtained by lithiation) is critical. At plant scale, in-line monitoring technique should be used to monitor the outlet concentration of both the reactions. A suitable ratio controller should be employed to control the volumetric ratio (hence the mole ratio) at a larger scale. The entire synthesis can be depicted in the form of a block diagram as shown in Figure 4A. The use of a Vapourtec platform brings a certain level of automation, however, a large variation in the synthesis conditions between two subsequent steps makes it not a fully automated protocol. To make it such a platform, in Figure 4B, we have depicted a process instrumentation diagram for this scheme and subsequently discuss the details.

The P&ID diagram in Figure 4B indicates that the flow rate of the Weinreb amide can be fixed at the desired set point using a control valve as it is the limiting reagent while the flow rate of Grignard’s reagent can be controlled using the ratio controller. Both the process streams can be preheated at the reaction temperature (i.e., 60 °C) using a feedback controlled heat-exchanger. The preheated streams have to be mixed in a suitable mixer followed by the reactor. The reactor can be maintained at the desired temperature using a jacket. The flow rate of the jacket fluid can be the manipulating variable to automate the synthesis that can have fixed conversion as a set-point. The outlet concentration or the conversion can be monitored online to check the variation around the set-point value. This process stream containing the ketone intermediate can be precooled to −50 °C. Aryl bromide and *n*-BuLi are precooled at −50 °C and reacted to obtain the lithiated product. The control strategy for lithiation reactor will be similar to the previous reactor. This lithiated intermediate and the ketone intermediate obtained by the simultaneous process can be mixed using a ratio controller at the optimum stoichiometric amount. The obtained lithium alkoxide can be monitored inline using a suitable analytical technique and the reactor control loop will be similar to previously discussed reactors. This process stream can be heated to ambient temperature using a heat exchanger before mixing it with trifluoroacetic anhydride whose flow rate can be controlled using a ratio controller. The intermediate obtained can be mixed with triethylamine using a ratio controller and can be preheated to 100 °C before passing through the reactor. Alternatively, both the process streams can be preheated separately before mixing. The concentration of the tamoxifen thus obtained can be monitored online. The whole system can be pressurized using a back pressure regulator to avoid any intermittent pressure variations due to phase change. It is possible to have a different P&ID structure depending upon the control variable at each stage. However, the merit of any such structure if implemented before optimizing the specific flow synthesis will help to generate very valuable data that can be used for transforming this chemistry into a process.

Lithiation reaction is highly exothermic and a special control strategy should be employed to avoid a runaway situation [44]. However, if a runaway event occurs an appropriate strategy should be developed to stop the reagents flow first and quench the reaction mass. More importantly, moisture sensors need to be installed on the system to avoid the possible contact of water with *n*-BuLi.

#### Case study 3: Multistep flow synthesis of rufinamide (telescopic synthesis)

**Figure 5:**
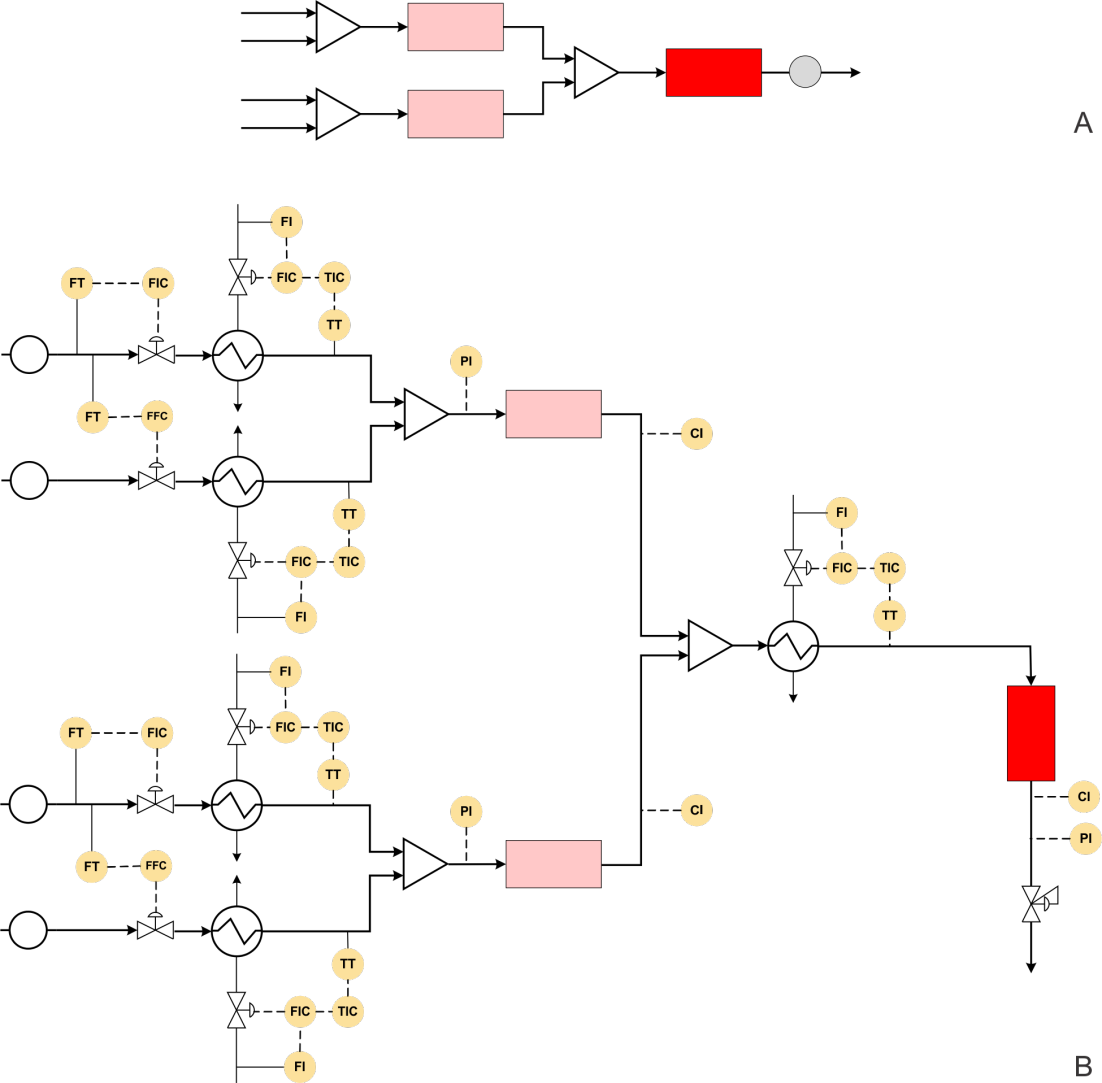
(A) Block diagram representation of the process shown in Scheme 3, (B) piping and instrumentation diagram of Scheme 3.

**Scheme 3:**
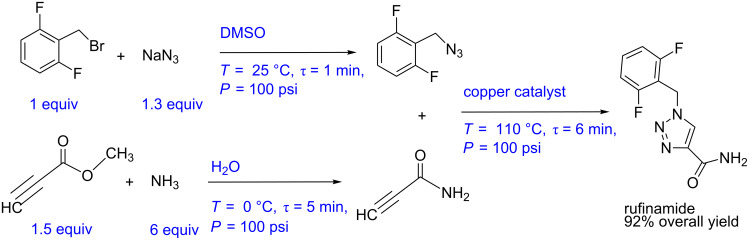
Multistep flow synthesis of rufinamide (Zhang et al. [14]).

Zhang et al. have reported the multistep synthesis of rufinamide, an antiepileptic agent [14] (Scheme 3). The process involves three steps namely azide synthesis, amide synthesis and click reaction or azide–alkyne cycloaddition. For the azide synthesis, the aryl bromide (1 equiv) and sodium azide (1.3 equiv) are reacted in a tubular reactor with 1 min residence time at 25 °C in DMSO medium. This resulted in quantitative yield. The amide synthesis is carried out simultaneously by mixing methyl propiolate (1.5 equiv) and aqueous ammonia (6 equiv) solution in a T-mixer followed by a coiled reactor. The authors report over 95% conversion at 0 °C for a residence time of 5 min. The products obtained by these two reactions are directly mixed and subsequently passed through the copper tube which acted as a catalyst for the cycloaddition reaction. At a temperature of 110 °C and a residence time of 6 min, rufinamide is obtained in 92% overall yield. The whole process is carried out under a pressure of 100 psi. The typical block diagram for this process is shown in Figure 5A, which depicts the simplicity of the experimental set-up for producing an expensive drug.

The block diagram, when transformed into a piping and instrumentation diagram, helps to know the possible automated flow synthesis platform with better clarity (Figure 5B).

For the said process the automated synthesis can be achieved as follows: The flow rate of aryl bromide can be fixed at a desired set point using a control valve or by setting the pump with feedback in the form of a pressure signal from the system. The flow rate of sodium azide has to be controlled using a ratio controller with respect to the pump for aryl bromide. Both the streams can be heated to the reaction temperature before mixing. This will help to save time in heating the reaction mixture and also to avoid undesired side reactions possibly due to prolonged contact of the reactants. The mixed stream has to be followed by a flow reactor with a jacket, where the jacket side fluid can be the manipulating variable whereas the outlet concentration of the reactor can be set as a control variable. Simultaneously methyl propiolate and aqueous ammonia can be precooled at 0 °C using a compact heat exchanger (it can even be a coil inside a jacket) before mixing at the desired molar ratio using a ratio control. The amide can be monitored at the reactor outlet using inline monitoring system (like UV, IR or Raman spectroscopy) which may be coupled with the reactor jacket flow rate to maintain the desired conversion. The azide and amide streams can be mixed and preheated at 110 °C. The preheated stream can then flow through a copper tubing reactor or a packed bed reactor with copper packings, whichever is scalable or easy to replace. The temperature of the reactor can be maintained by manipulating the jacket fluid flow rate. The rufinamide thus obtained can be monitored online. A back pressure regulator can be used to pressurize the entire reactor system.

#### Case study 4: Multistep synthesis for (±)-oxomaritidine (gas–liquid–solid reaction)

**Figure 6:**
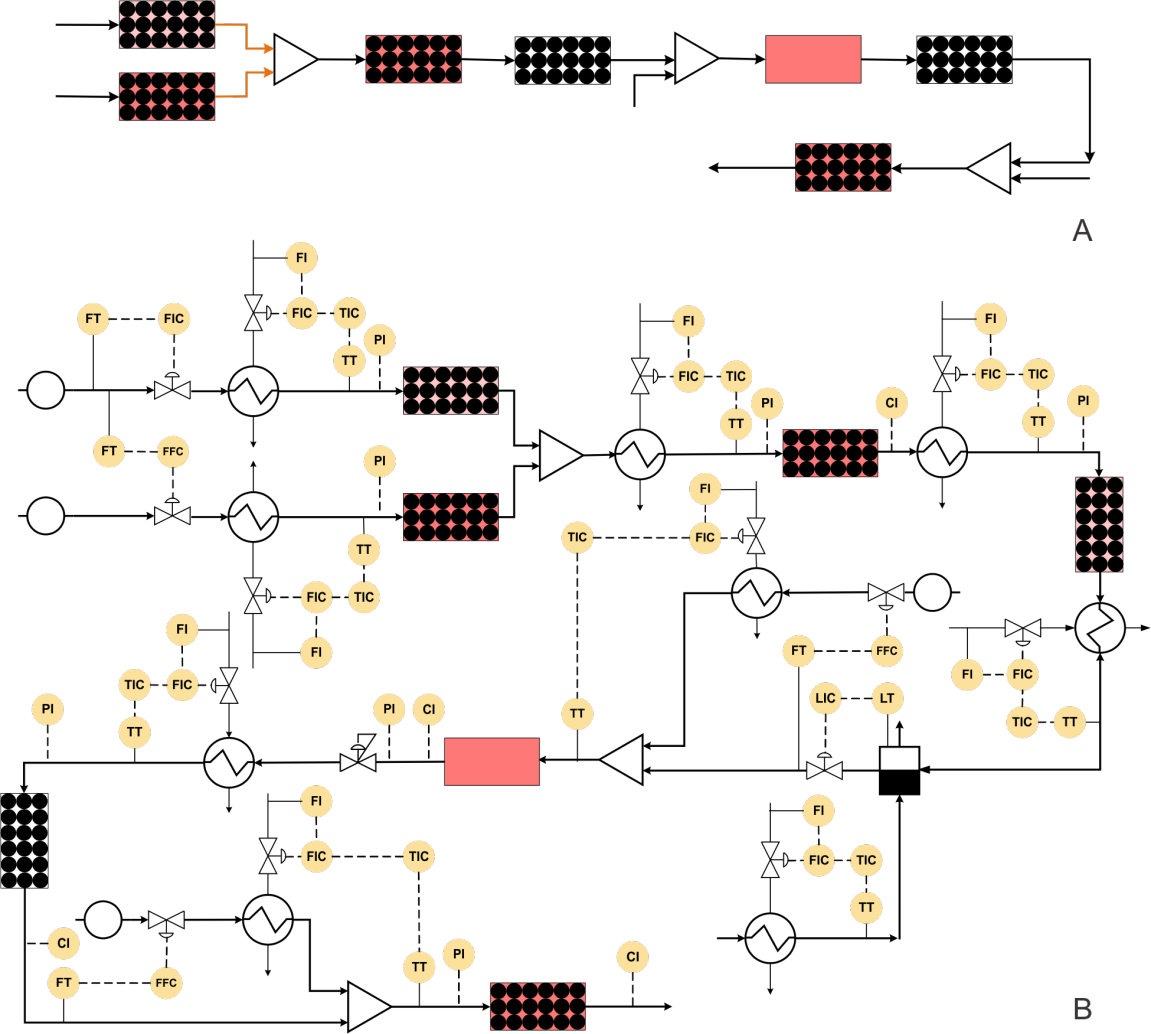
(A) Block diagram representation of the process shown in Scheme 4, (B) piping and instrumentation diagram of Scheme 4.

**Scheme 4:**
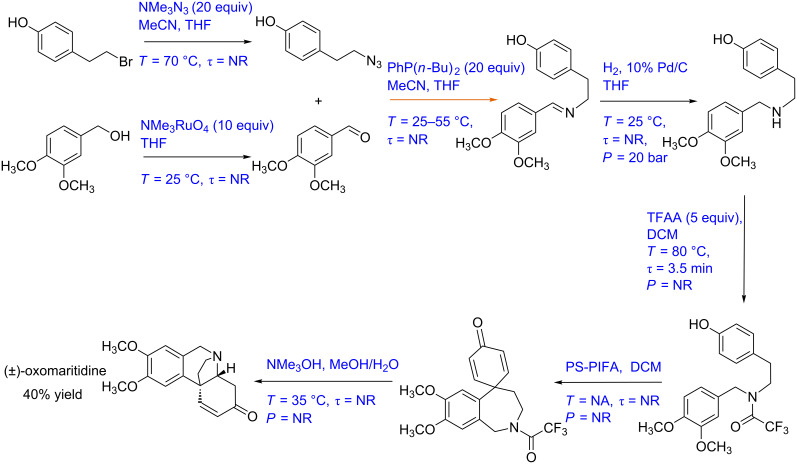
Multistep synthesis for (±)-Oxomaritidine (Baxendale et al. [9]).

Baxendale et al. have developed a multistep synthesis for (±)-oxomaritidine, a cytotoxic alkaloid of the amaryllidaceae family of natural products [9] (Scheme 4). The process involved seven reaction steps out of which the first two reactions were carried out in parallel. In the first step, 4-(2-bromoethyl)phenol is converted to its azide derivative by passing it through a glass reactor packed with azide exchange resin (20 equiv) at 70 °C to achieve quantitative yield. MeCN and THF were used as solvents. Dimethoxybenzyl alcohol (in THF) is oxidized to the corresponding aldehyde by passing it through a packed column containing tetra-*N*-alkylammonium perruthenate (10 equiv) at room temperature to achieve quantitative yield. Further, these two products are reacted with each other to get the imine intermediate. The catch and flow technique is used with polymer-supported phosphine (20 equiv) as the trapping agent. The imine is further hydrogenated at 25 °C and 20 bar pressure by using an H-cube reactor with 10% Pd/C as a catalyst [72]. Trifluoroacetylation of the amine intermediate is then carried out in a chip reactor with trifluoroacetic anhydride (in DCM) as a reagent. The reaction temperature and residence time are 80 °C and 3.5 min, respectively. This product further undergoes a coupling reaction in a packed column containing polymer-supported [bis(trifluoroacetoxy)iodo]benzene (PS-PIFA), which yields a seven-membered tricyclic intermediate with 50% yield. The tricyclic intermediate is further mixed with MeOH and water (4:1) and passed through a packed column containing a polymer-supported base at 35 °C. The target compound (±)-oxomaritidine was obtained in 40% yield. The block diagram of different steps performed in this synthesis is shown in Figure 6A. This is a relatively simple approach for a complex transformation. However, there are complexities in terms of the difference in the reaction conditions at each step, where one has to ensure that unreacted reactants do not enter the next step and the heating/cooling rates are managed efficiently to avoid a longer residence time during automation.

The corresponding P&ID diagram and associated complexities that one would need to deal for automating such a synthesis are discussed below. Figure 6B shows the P&ID for the (±)-oxomaritidine manufacturing process. The flow rate of the limiting reagent, 4-(2-bromoethyl)phenol can be fixed at a desired set-point using a control valve. This process stream can be preheated at the reaction temperature (70 °C). It can then be passed through the reactor packed with azide exchange resin maintained at the desired temperature using a jacket. The azide exchange resin will get consumed after some time and the corresponding section needs to be activated or recycled in order to maintain continuous production. In such cases, it is possible to have two parallel reactors containing a packing of azide exchange resin, which can be operated in a cyclic manner to maintain continuous flow or an efficient arrangement of continuous activation of the bed like a simulated moving bed chromatographic reactor (SMB). Alternatively, one can also charge the azide resin as a suspended mass in the flow to avoid this complexity to some extent and to have a filter to keep the resin retained in the reactor. Considering the existing configuration of the packed bed reactor with the cyclic operation, dimethoxybenzyl alcohol can be preheated and passed through the packed bed reactor containing the oxidizing reagent as packing material to obtain the corresponding aldehyde. The packed bed reactor control strategy will remain identical for all reagent packed reactors. The azide and the aldehyde intermediate can be mixed and preheated at the reaction temperature and passed through a phosphine-functionalized polymer packed bed reactor to obtain the imine intermediate. This imine intermediate can be reacted with H_2_ in a commercial reactor with an integrated control system. After hydrogenation, the solvent switch can be carried out in an evaporator by removing the THF solvent and then re-dissolving the intermediate in DCM as solvent. The outlet flow rate of the evaporator can be controlled by maintaining a fixed liquid level inside the evaporator. Trifluoroacetic anhydride can be preheated and mixed with the amine intermediate stream and passed through the reactor. The reactor is maintained at the desired temperature using a jacket. The outlet concentration of the reactor can be measured inline and the reactor jacket fluid flow rate should be manipulated to maintain a steady state at the reactor outlet. The process stream can be passed through the heat exchanger to reach the reaction temperature before passing through the packed bed reactor containing polymer-supported [bis(trifluoroacetoxy)iodo]benzene as packing material. The control strategy for the packed bed reactor will be similar as discussed earlier. The process stream containing the tricyclic intermediate can be cooled to 35 °C by mixing a cold stream of MeOH/water at the desired mole ratio using a ratio controller. The mixed stream can be passed through a packed bed reactor containing base to obtain (±)-oxomaritidine. The outlet concentration of (±)-oxomaritidine can be monitored online. Along with the concentration, it is also necessary to monitor the mass flow rate at the outlet to ensure that the reactions and conversions in the entire system are as per the design. The flow regimes in the packed bed reactor described here for liquid–solid reactions will be different based on wettability and such considerations need to be evolved separately as they become rate controlling when one goes for scale-up.

#### Case study 5: Multistep synthesis for ibuprofen (low overall residence time)

**Figure 7:**
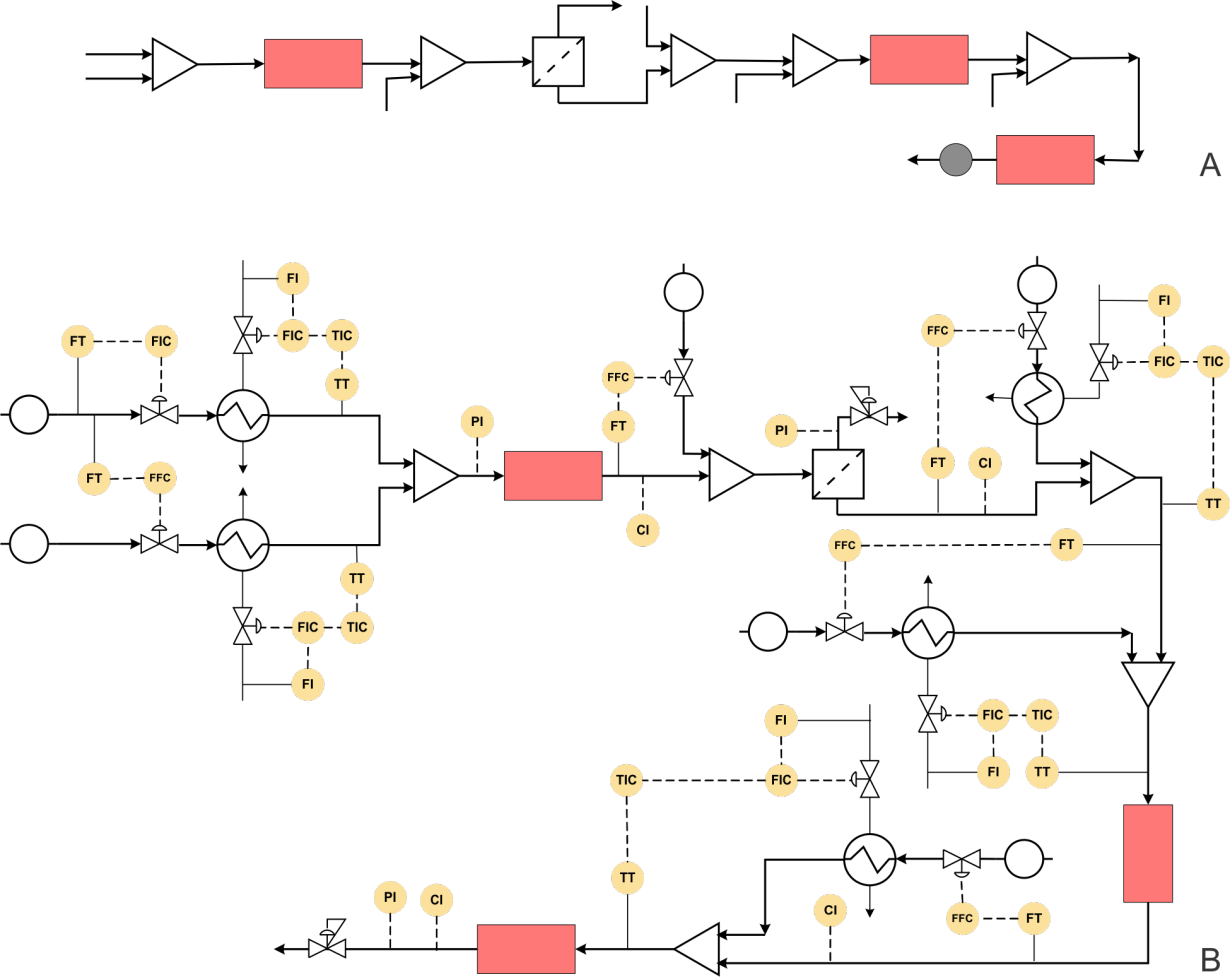
(A) Block diagram representation of the process shown in Scheme 5, (B) piping and instrumentation diagram of Scheme 5.

**Scheme 5:**
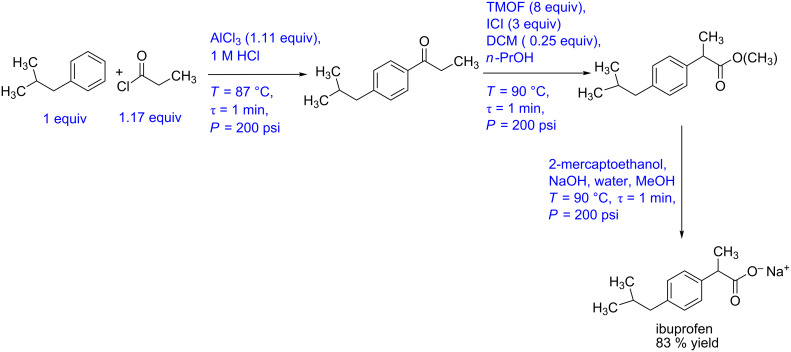
Multistep synthesis for ibuprofen (Snead and Jamison [60]).

In a fascinating approach, recently Sneed and Jamison have reported a multistep synthesis for ibuprofen with a total residence time of the entire process approximately equal to 3 minutes [60] (Scheme 5). The process involves three reaction steps and one separation step. In the first step, a Friedel–Crafts acylation of isobutylbenzene (1 equiv) and propionyl chloride (1.17 equiv) in the presence of AlCl_3_ as Lewis acid was carried out in a tubular reactor. The residence time is one minute, and the temperature is maintained at 87 °C. The outlet of the reactor is mixed with aqueous HCl, and the organic and aqueous streams were separated by using an inline membrane separator. The ketone derivative was obtained in 95% yield (measured at the outlet of the membrane separator). This aryl ketone intermediate is mixed with trimethylorthoformate (8 equiv) in DMF solution and ICI as the promoter (3 equiv) in *n*-PrOH and was subjected to an oxidative 1,2-aryl migration. The reaction is carried out in a coiled reactor at 90 °C and 1 min residence time. The outlet stream is subjected to an alkaline solution of 2-mercaptoethanol, which quenched the ICI and carried further saponification of the ester intermediate in another tubular reactor at 90 °C and 1 min residence time. The entire process is carried out at 200 psi pressure and the yield of the target product ibuprofen is reported to be 83%. This report has been among the most eye-popping works in the recent time. This is largely because of the common usage of this medicine in huge volumes across the globe. As compared to the existing conventional process for ibuprofen, if this approach is to be followed right up to production scale, it needs a very different approach (while keeping the synthesis pathway unchanged). In order to have a first cut analysis of what that approach would involve if the process is to be optimized, in the below we give the synthesis pathway in terms of a block diagram (Figure 7A) that is easy to interpret and then evolve a piping and instrumentation diagram that will allow generating necessary data leading to scale-up. Figure 7B shows the P&ID for the ibuprofen manufacturing process. The flow rate of the limiting reagent, isobutylbenzene can be fixed at the desired set point using a control valve. The flow rate of the propionyl chloride stream can be controlled by using a ratio controller.

Both these streams should be preheated at 87 °C using a heat exchanger with feedback control. The preheated streams can be mixed in a reactor whose temperature can be controlled by a jacket. The outlet concentration of the intermediate can be monitored online and accordingly the jacket fluid flow rate should be varied for maintaining a steady state. A stream of aqueous HCl is mixed with this process stream using a ratio controller. The aqueous and organic phases will separate in the membrane separator. The back pressure regulator can be installed on the aqueous stream to create the desired pressure and facilitate complete separation. Trimethylorthoformate and ICI promoter also need to be preheated to 90 °C and mixed with the process stream containing the aryl ketone intermediate. The reactor can be maintained at the desired temperature using a jacket or tube-in-tube approach. The concentration of the ester intermediate can be monitored using the suitable inline analytical technique. The reactor jacket flow rate can be varied to control the intermediate ester concentration thereby ensuring that the reactor temperature is within the set-point and does not lead to side products. This stream can be mixed with a preheated alkaline 2-mercaptoethanol stream using another ratio controller to meet the stoichiometry. The combined stream can pass through a jacketed reactor, and the outlet concentration of ibuprofen can be monitored inline. Once again, as mentioned previously, the jacket fluid flow rate can be used as a manipulating variable for controlling the reactor conversion and selectivity.

#### Case study 6: Multistep synthesis of cinnarizine, cyclizine, and buclizine derivatives (inline quenching)

**Scheme 6:**
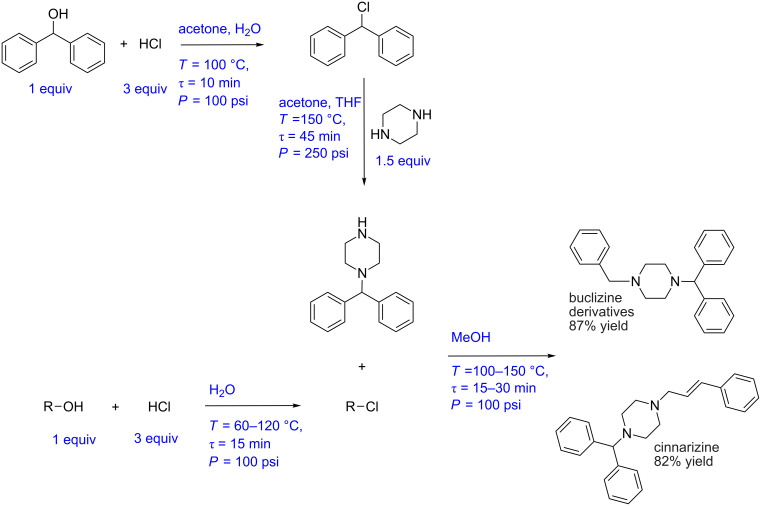
Multistep synthesis for cinnarizine and buclizine derivatives (Borukhova et al. [23])

Borukhova et al. have reported a multistep synthesis for cinnarizine, cyclizine, and buclizine derivatives [23]. These drugs belong to the antihistamine family. The process involves 4 reaction steps and two liquid–liquid extraction steps (for cinnarizine and buclizine derivatives). In the first step diphenylmethanol (1 equiv) is mixed with HCl (3 equiv) and passed through a tubular reactor at 100 °C and 10 min residence time. An acetone and water mixture is used as solvent and the reactor is pressurized at 100 psi using a back pressure regulator. The resulting aryl chloride is obtained in 97% yield. The excess HCl is then quenched with NaOH and the process stream is passed through the membrane separator. The outlet pressure of the aqueous stream was maintained at 2 psi pressure resulting in a perfect separation. The aryl chloride is further reacted with piperazine (1.5 equiv) to obtain 1-(diphenylmethyl)piperazine in 92% yield. The optimum conditions were 150 °C, 45 min, and 250 psi. The alcohol substrate is then reacted with HCl in a tubular reactor in parallel to get the corresponding aryl chloride. The temperature range was 60–120 °C for different substrates whereas the residence time and pressure were maintained at 15 min and 100 psi, respectively. The excess HCl was quenched with NaOH, and the organic phase was separated using a membrane separator. Aryl chloride is then mixed with 1-(diphenylmethyl)piperazine (obtained from the previous step) and methanol and passed through a tubular reactor maintained at 100–150 °C, over 15 to 30 min and at 100 psi pressure. The target drugs cinnarizine and buclizine derivatives are obtained in 82% and 87% yield, respectively (Scheme 6). This process is relatively simple yet involving the use of in-line extraction and separation, which would have very different separation time scales when compared to the reaction time scale. Developing an automated platform for such a synthesis is indeed a challenge. In the below, we describe this approach in a way that can help to build an automated synthesis platform.

**Figure 8:**
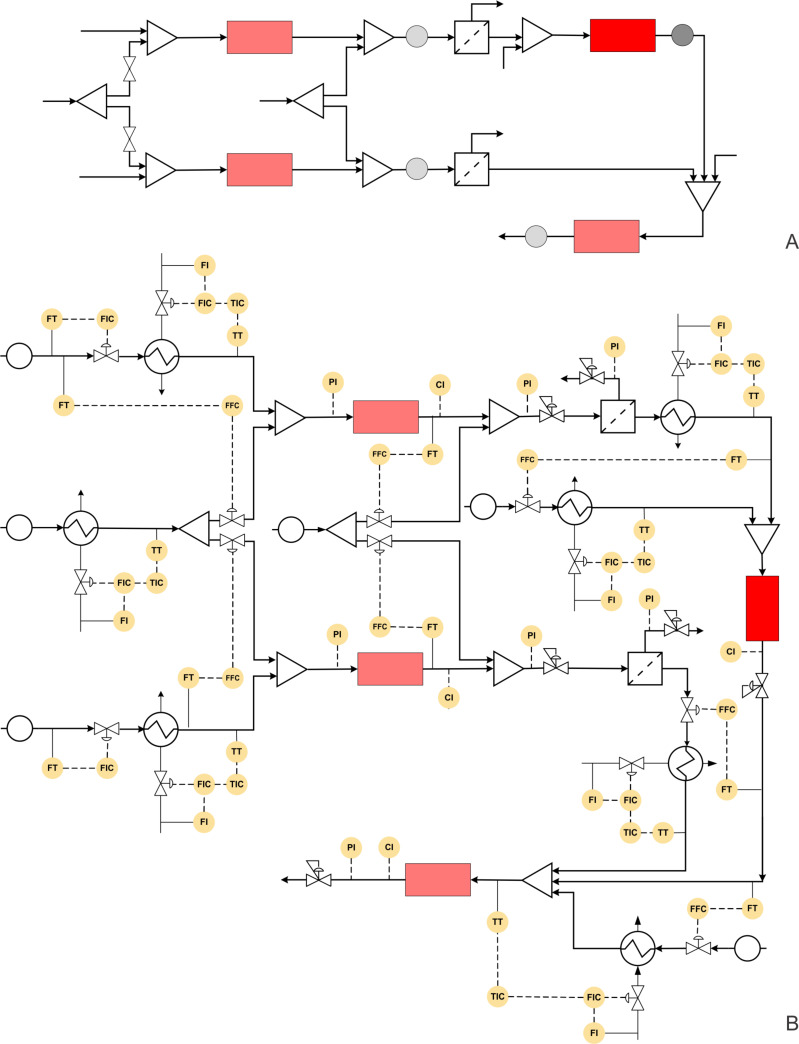
(A) Block diagram representation of the process shown in Scheme 6, (B) piping and instrumentation diagram of Scheme 6.

Figure 8A and 8B show the block diagram and possible P&ID for the cinnarizine/buclizine derivative manufacturing process. Initially, the flow rate of diphenylmethanol and the alcohol derivative should be fixed at the desired set point using a control valve. These streams can be preheated using a heat exchanger with feedback control. Aqueous HCl can also be preheated to the reaction temperature by applying suitable back pressure, and the stream can be split into two streams with a ratio controller for both the streams. Both the alcohol substrates can be mixed to react with HCl in the separate jacketed reactor to produce the corresponding aryl chlorides. The aqueous NaOH stream can be split into two streams (similar to the HCl stream discussed previously) and mixed with the reaction stream to quench the reaction. Alternatively, an inline pH flow cell can be used to measure the pH of the quenched solution and to send a feedback signal to control the flow rate of the NaOH solution [24]. After quenching the reaction, the aqueous and organic phases can be separated using membrane separators. The pressure at the aqueous outlet can be controlled using a back pressure regulator to achieve the desired degree of separation. However, the separator needs to be designed to match the production capacity as it comes from the outlet of the reactor. Moreover, the separator needs to have a pressure transmitter to measure the pressure drop across the membrane to ensure that for higher or lower pressure drop values than the set-point values, an early indication of blocking or wearing of the membrane is given. Piperazine can be preheated and mixed with aryl chloride (obtained from diphenylmethanol) using a ratio controller to maintain the desired mole ratio. The mixed streams should be passed through the jacketed reactor with a jacket flow rate as the manipulating variable and the reactor outlet concentration as a controlled variable. The obtained 1-(diphenylmethyl)piperazine can be mixed with aryl chloride (obtained earlier) and with preheated MeOH at the desired mole ratio using a ratio controller. The mixed stream can then be passed through a jacketed reactor. The control strategy for the reactor can be similar to the above discussed reactor. The concentration of the API, cinnarizine/buclizine can be monitored in real time using an appropriate inline analytical technique. A back pressure regulator can be used to pressurize the entire system. However, if the membranes in the separators do not withstand these operating pressure for the reaction, one must isolate the zones of different pressure. Also, for the corrosive segments while PTFE or other commonly used flexible tubes would work at laboratory scale, these may not withstand pressure and hence it is advisable to use non-corrosive hastelloy or tantalum lined tubes or glass reactors that can withstand the process pressure.

#### Case study 7: Multistep synthesis of (*S*)-rolipram (gas–liquid–solid reaction)

**Scheme 7:**
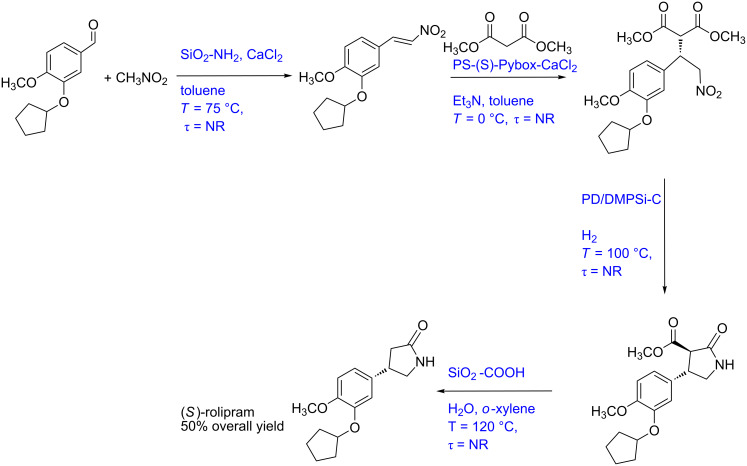
Multistep synthesis for (S)-rolipram (Tsubogo et al. [4])

**Figure 9:**
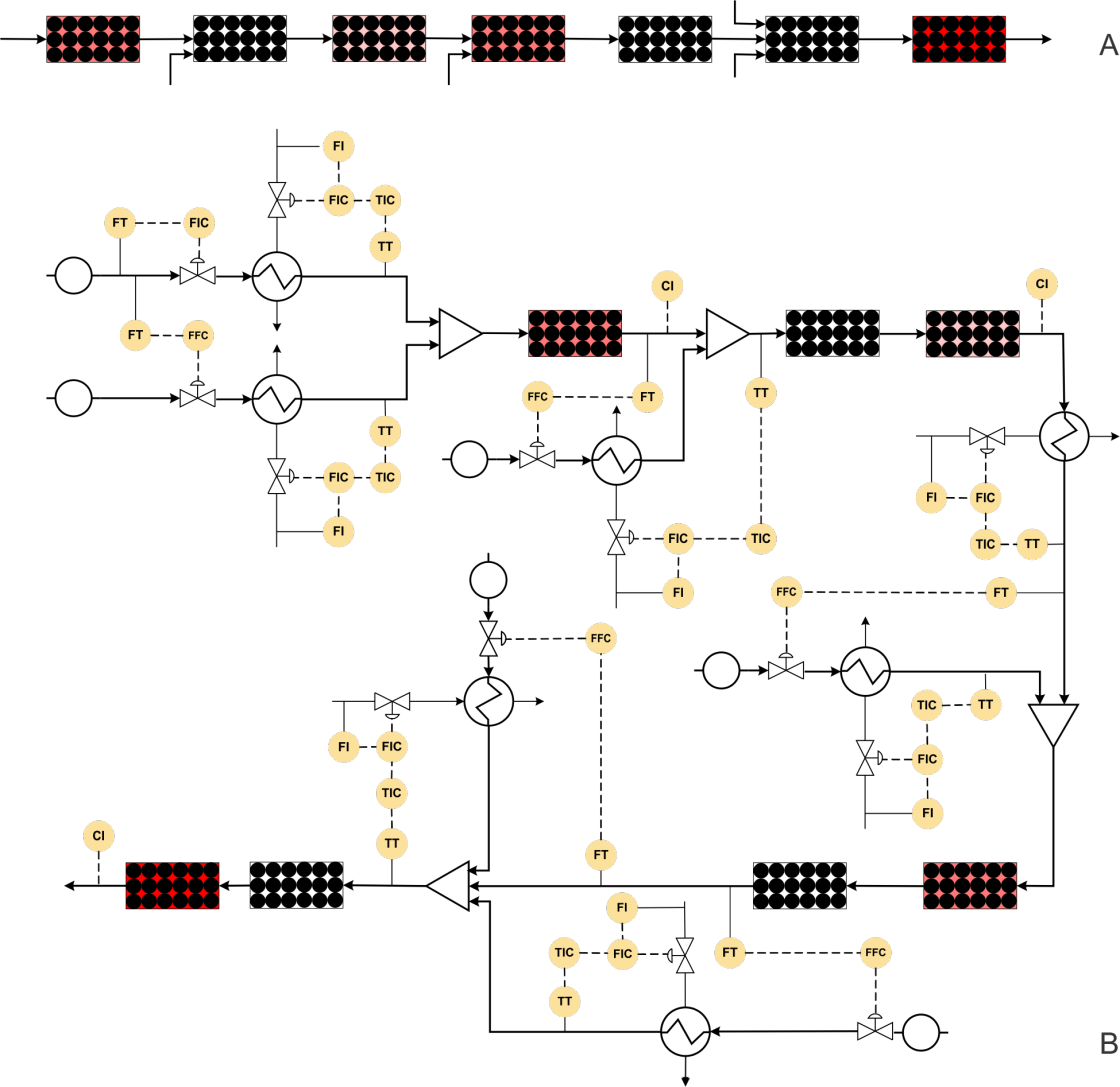
(A) Block diagram representation of the process shown in Scheme 7 (colours for each reactor shows different reactor temperatures), (B) piping and instrumentation diagram of Scheme 7.

Tsubogo et al. developed a multistep synthesis of (*S*)-rolipram, a drug belonging to the GABA family (Scheme 7) [4]. This is an excellent example for the use of several adsorption columns to isolate impurities. This work is a lucrative approach for the end-to-end synthesis of high value drugs. However, using so many packed beds is a challenging task when it comes to scale-up where the feed-back and feed-forward effects of individual packed beds. Before raising more operational complexities in scaling-up this approach, here we briefly describe the synthesis method. In a first step, a solution of aldehyde and nitromethane in toluene is passed through a packed column containing SiO_2_-NH_2_ and CaCl_2_ as a catalyst and was maintained at 50 °C. The intermediate nitroalkene is obtained in 90% yield. A solution of malonate and triethylamine in toluene is mixed with the nitroalkene stream and passed through a packed column containing MS 4 Å to obtain stability in the system. This process stream is then passed through a catalytic reactor packed with polymer-supported (S)-pybox–calcium chloride maintained at 0 °C. The Michael addition product is obtained in 84% yield which was subsequently reacted with hydrogen in a catalytic reactor containing Pd/DMPSi-C as the catalyst. The optimal operational conditions for the hydrogenation were 100 °C at atmospheric pressure. The γ-lactam was obtained in 74% yield. In the final stage, this product is hydrolysed and decarboxylated by passing it through a reactor containing silica-supported carboxylic acid at 120 °C. The final overall yield of the product (*S*)-rolipram is reported to be 50%. This synthesis method looks to be the cleanest approach so far as it uses multiple reactors for individual transformations. Figure 9A shows the block diagram of this synthesis protocol, which actually brings out many challenges for scale-up for this process. In Figure 9B we have shown the P&ID of a possibly automated process for the synthesis of rolipram.

Initially, the flow rate of the aldehyde substrate should be fixed at the desired set point using a control valve while the nitromethane flow rate should be controlled using a ratio controller. Both these process streams should be preheated in a heat exchanger with a feedback controller. The mixed streams can then pass through a catalytic packed bed reactor with a jacket to maintain the reaction temperature. The intermediate nitroalkene can be monitored at the reactor outlet, and the jacket fluid flow rate can be manipulated accordingly to maintain a steady state. Typically, for a catalytic reaction in a fixed bed reactor, the temperature profile is not uniform over the cross-section and thus can result in variation of the selectivity. The effect can be minimized by using a multi-tubular fixed bed reactor of smaller tube diameter, provided the flow is uniformly distributed in each tube. The malonate and triethylamine stream can be pre-cooled and mixed with the nitroalkene stream. The mixed stream can be passed through a packed bed reactor containing the catalyst maintained at 0 °C using a cooling jacket. The Michel addition product obtained can be monitored inline and the concentration can be controlled by varying the jacket flow rate. This process stream can be mixed with preheated hydrogen gas using an appropriate ratio controller. This mixed stream can then be passed in a packed bed reactor containing the Pd catalyst and maintained at 100 °C using a heating jacket. Since the temperatures are different for subsequent reactions, the issues related to conjugate heat transfer and reaction progress in the connection section needs to be carefully analyzed. In the case of deviations from the exact or desirable residence time and for the cases where the residence time distribution is non-Gaussian or Gaussian RTD with wider time scale, the formation of impurities and their carry-forward to the next reactor can be detrimental to the process. A systematic model needs to be developed to quantitatively obtain the yields of the products and impurities at different locations spatially and at different scales. The concentration of the hydrogenated product can be monitored and controlled by manipulating the jacket fluid flow rate. This process stream can be mixed with a preheated *o*-xylene and water stream and passed through a packed bed reactor containing celite which can act as a filter medium. The mixed stream can then be passed to a packed bed reactor containing silica-supported carboxylic acid and was maintained at 120 °C using a heated jacket. A similar control strategy as for the packed bed reactor can be employed. It needs to be realized that since the reaction temperature in each fixed bed is different, an in-line heater is needed wherever necessary so that either quenching of reactions or sudden changes in the conditions can be avoided.

#### Case study 8: Multistep synthesis for amitriptyline (gas–liquid reaction)

**Scheme 8:**
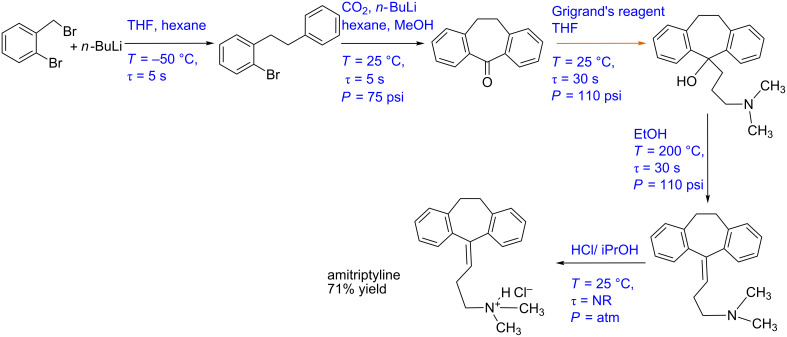
Multistep synthesis for amitriptyline (Kupracz and Kirschning [7]).

Kupracz and Kirsching have reported a continuous multistep synthesis approach for amitriptyline, an antidepressant drug (Scheme 8) [7]. The process involves six reaction steps. Initially, a lithiation/Wurtz coupling reaction was carried out between benzyl bromide (in THF) and *n*-BuLi (in *n*-hexane) in a coiled steel reactor (1 mm ID and 0.5 mL) at −50 °C and 5 s residence time. This crude mixture of aryl bromide was reacted with CO_2_ in a tube-in-tube reactor at −50 °C followed by a PFA reactor coil (0.8 mm ID and 0.5 mL) where the carboxylation took place at 25 °C. After removing the unreacted gas the reaction mixture was mixed with *n*-BuLi (in *n*-hexane). A Parham cyclization is carried out in 0.5 mL PFA reactor coil (0.8 mm ID) at 25 °C to yield 76% of ketone intermediate. This product is dissolved in MeOH and was isolated. This product is dissolved in THF and reacted with the Grignard reagent in a 0.5 mL PFA coil reactor (0.8 mm ID) at 25 °C and 30 s residence time. The crude product is protonated with EtOH and subjected to water elimination. The water elimination took place at 200 °C (using inductive heating) and 30 s residence time in a packed reactor column. The process fluid is cooled to room temperature using a heat exchanger and was reacted with HCl (in isopropanol) which gives the corresponding salt. This was further recrystallized from EtOH/Et_2_O to yield the amitriptyline hydrochloride salt (71%).

Interestingly, the authors have used the tube-in-tube system in series with a coiled reactor for the carboxylation step. While such systems do work for very small scale, the tube-in-tube approach is not easily scalable as the reaction rates enhanced due to higher mass transfer rates at the beginning of the tube would decrease subsequently making it complex to design a reactor for large-scale production. A simple gas–liquid slug flow in the coiled reactor should work. Moreover, it is easy to maintain the mole ratio of CO_2_ and reactant in the coiled reactor. By selecting an appropriate flow regime, one can maximize the mass transfer rate and hence optimize the reaction. Using very different solvents throughout the process viz. THF, *n*-hexane, methanol, ethanol, isopropyl alcohol and Et_2_O will increase the downstream separation cost. We selected this process as it uses a tube-in-tube reactor for gas–liquid reaction along with a complex combination of solvents. Such an approach is going to be challenging for scale-up and specific variations in the process are definitely needed to make it automated. The automation proposed in the rest of this case study is only one such alternative and it will depend upon the choice of flow reactor.

**Figure 10:**
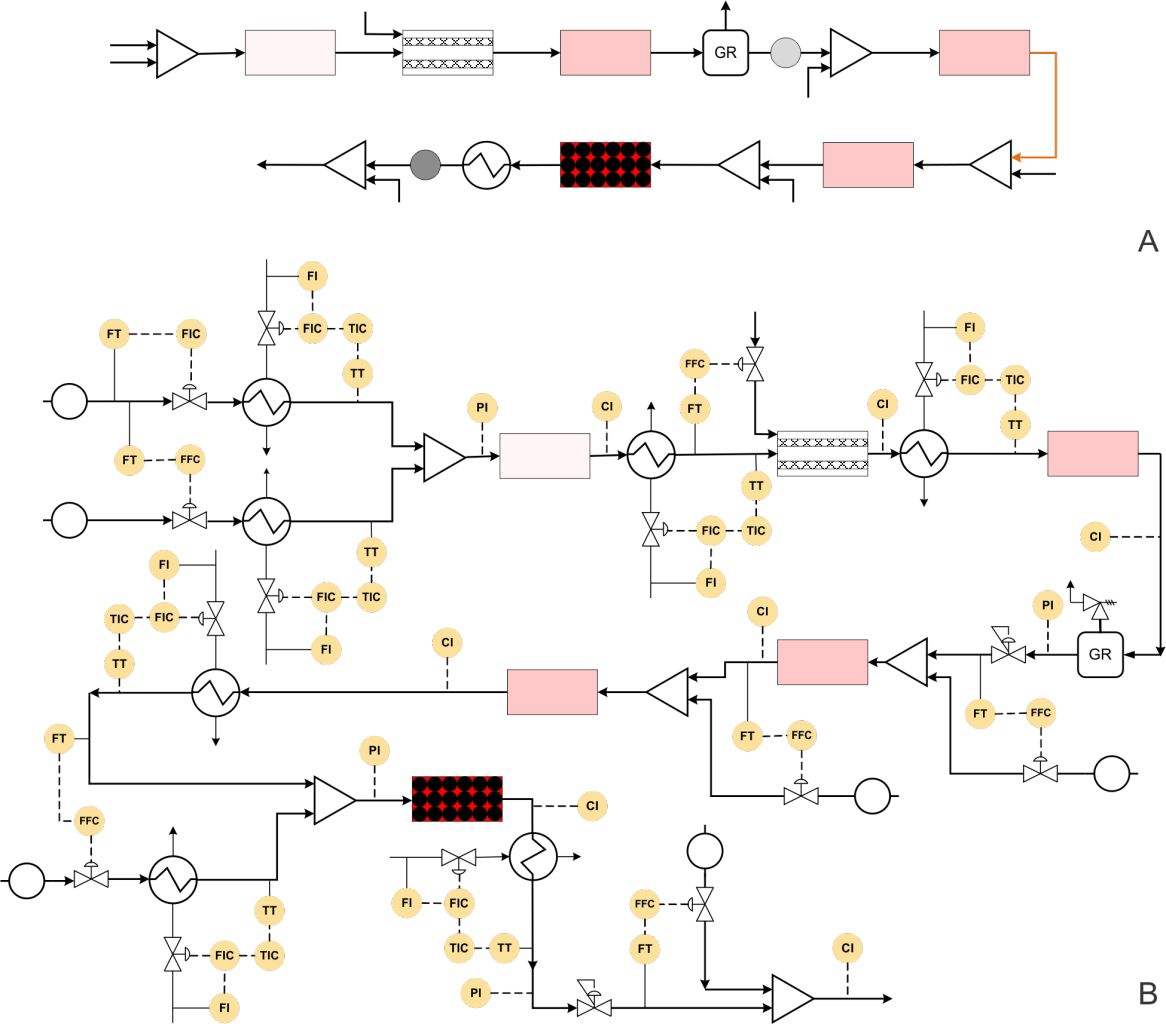
(A) Block diagram representation of the process shown in Scheme 8, (B) piping and instrumentation diagram of Scheme 8.

Figure 10A and 10B shows the block diagram and P&ID for the amitriptyline manufacturing process. The mole ratio of benzyl bromide and *n*-BuLi can be controlled using a suitable ratio controller. The flow rate of benzyl bromide can be fixed at a suitable set point value depending on the scale of operation as it is the limiting reagent. Both these process streams can be pre-cooled separately using heat exchangers. For the heat exchanger, the outlet temperature of the process stream will be the controlled variable while the flow rate of the coolant will be the manipulating variable. After precooling, the reactants can be mixed in a suitable mixer and allowed to react in the reactor. The temperature of the reactor can be controlled by the jacket containing coolant (not shown in the Figure). The outlet concentration of the products can be monitored using a suitable inline analytical technique. In this case, the concentration of the reactor outlet can be the controlled variable and the coolant flow rate of the reactor jacket should be the manipulating variable. Alternatively, it is also possible to control the reactor outlet temperature by manipulating the jacket coolant flow rate. The crude mixture can be precooled again as it will be at relatively higher temperature due to absorption of the exothermic heat. This crude mixture can be passed through a heat exchanger to cool it and then passed through a membrane reactor. Using an appropriate ratio controller the flow rate of the CO_2_ has to be controlled while measuring the flow rate of the crude mixture. The reaction mixture at the outlet can be heated to ambient temperature using a heat exchanger followed by a reactor. The outlet conversion of the reactor has to be monitored using a suitable inline analytical technique and the reactor temperature should be manipulated to control the outlet conversion. The excess CO_2_ can be removed via a gas–liquid separator followed by a gas release valve attached to a pressure regulator. The ratio controller should control the molar ratio of the carboxylated intermediate and the *n*-BuLi. The concentration of the Parham cyclization product can be monitored and controlled at the reactor outlet by manipulating the coolant flow rate of the reactor jacket (not shown in the Figure). The process stream can be further mixed with the Grignard reagent in the desired stoichiometric ratio and passed through the reactor. The reactor temperature is maintained at ambient conditions using a cooling jacket. The concentration of the intermediate is monitored at the reactor outlet and the control action (flow rate of jacket coolant) can be taken accordingly. The process stream can be preheated using a heat exchanger and by mixing with preheated EtOH. Heating oil or high-pressure steam should be employed as a heat exchanger utility as higher temperatures (200 °C) are required. The process stream after passing through the reactor should be cooled at ambient temperature using a heat exchanger. The cooled process stream then can be mixed with HCl at suitable stoichiometry using a ratio controller to get the hydrochloride salt of amitriptyline. The back pressure regulator can be used to pressurize the system at the desired set point.

### Examples of laboratory scale automated syntheses

Recently few excellent reports have appeared in the literature where control strategies are implemented at laboratory or bench scales. Saleemi et al. have investigated the effect of different control strategies on crystallization processes [73–74]. Process analytical technologies can be used to monitor concentrations, particle shape and size, and to control the temperature in crystallization processes [73–76]. More details about in-line monitoring techniques and control strategies in the industrial crystallization process can be found in the recent reviews [77–79]. Johnson and co-workers have demonstrated a controlled large-scale continuous-flow synthesis for various processes viz. asymmetric hydrogenation [80], direct asymmetric reductive amination [81] and asymmetric hydroformylation [82] and continuous Ir-catalyzed homogeneous reductive amination reaction [83]. Poh et al. demonstrated a multistep flow synthesis of pyrazole derivatives [84]. The process involved three reactions namely diazotization, reduction, and a hydrolysis/cyclocondensation. In-line flow IR spectroscopy was employed to monitor the concentration of the diazonium salts and the desired carbonyl intermediate. Computer integration with in-line IR and pumps also facilitated the control of the flow rate of the pentane-2,4-dione and HCl for the final hydrolysis/cyclocondensation step. In one of the most sophisticated systems, Adamo et al. have reported a compact (1.0 m (w) × 0.7 m (l) × 1.8 m (h)) and reconfigurable system capable of synthesizing and formulating various active pharmaceutical ingredients [3]. The system had a reconfigurable upstream unit which included a feed reservoir, reactors, pumps, separators and back pressure regulators. The upstream unit was followed by a downstream unit capable of further purification like tanks for precipitation of APIs, crystallizers, filters, etc. Strategic locations in the process were employed with suitable sensors for measuring the temperature, pressure, level and flow and were coupled with data acquisition systems for real-time monitoring. An inline attenuated total reflection (ATR) Fourier transform infrared (FTIR) system (FlowIR) was also employed for online monitoring of the formed APIs namely diphenhydramine hydrochloride, lidocaine hydrochloride, diazepam, and fluoxetine hydrochloride.

Hartman et al. have designed a microfluidic distillation operation and integrated it with a multistep reaction and liquid–liquid extraction [30]. The authors have used a compression chuck that controlled the inlet and outlet temperature of the microreactor and other components using a Thermo Scientific NESLAB RTE-7 refrigerating bath. For controlling the device temperature the authors employed an Omega 120V cartridge heater controlled by an Omega CN9000 series PID controller.

While these case studies are an encouraging sign of taking flow synthesis one step ahead, automation also faces challenges, often from the complexity of integrating various synthesis steps and variability in chemistry including phases of reactants, products, byproducts and catalysts. In the next section of this review, we have highlighted a few of such challenges that one might have to critically review before moving for automation.

### Challenges in automation

**Challenges to be addressed before automating any process:** The discussion so far brings out the approaches for transforming specific continuous synthesis methods to a possible automated platform. There are a few common techniques and tools used in flow synthesis, which can face challenges when automating as well as during the scale-up. Table 1 shows the challenges that need to be addressed before automating any process at pilot scale so that the necessary care can be taken at the laboratory scale to avoid such issues, which can make a route completely unviable. Secondly, safety becomes a major concern during scale-up and the information desired to check the issues relevant to hazard and safety of a synthesis route and conditions has to be monitored right from the laboratory scale synthesis. Thus, creating an automation platform for the specific synthesis is not sufficient but it is absolutely necessary to check the safety of the entire process based on the conditions, reactants, products and their stability.

**Table 1:** Common challenges that need to be addressed before automating any process.

Challenges	Comments

Induction heating [10]	• Additional cooling system may be required for cooling the reactor or distillation units. • Will increase the overall cost. • The control system can be complex due to different response times of heating and cooling cycles.

Increasing flow rate from inlet to the final outlet	• The flow rate at subsequent reactors will increase and hence a larger volume reactor will be required to maintain the required residence time. • Set-points for each reaction step will be different and need a different control structure.

Axial dispersion [85]	• Increasing flow rate along the synthesis path will increase the axial dispersion resulting in relatively lower conversions. • Additional volume should be provided for the reactor to overcome the effect of dispersion.

Number of mixers/ joints	• In multistep synthesis, there are relatively more T-joints/mixer which will cause pressure drop. • The control valves will contribute to significant pressure drop.

Material of construction	• At pilot scale, the process will run for a relatively larger time and hence reagents can corrode the reactors/pipelines. • Selecting appropriate material of construction becomes critical before automating any process at pilot scale.

Table 2 shows the different variables or parameters involved in any reaction or separation processes. Knowing the exact value of these variables like temperature, residence time and pressure is essential for obtaining reproducible experimental results. The reagents are often required to be preheated or precooled if there is a significant difference in the reactor temperature and the ambient temperature. Preheating can be done by simply using a tubular reactor or using a suitable heat exchanger. Preheating or precooling should always be done before mixing reagents. If the reagents are subjected to any reactor maintained at a certain constant temperature (like a thermostat or temperature bath) without preheating or precooling, there can be a noticeable temperature profile in the reactor. This temperature profile can largely contribute to the conversion and selectivity of the reaction under consideration. In such cases the experimental yield is highly sensitive to the temperature profile and thus preheating or precooling should be opted to minimize this sensitivity and make the process more robust. It should be clearly mentioned whether the reported temperature is of the reactor/temperature bath or the process stream. The temperature of the reactor surface and the process fluid can be significantly different in some cases [10]. Residence time is an important time scale for designing any reactor. Residence time along with different time scales like mass transfer, mixing, heat transfer and dispersion are useful in finding the controlling step [86]. This helps in selection of the appropriate reactor device for pilot or commercial scale operations. Surprisingly very few researchers have reported the residence time for a packed bed type reactor [28]. For calculating the residence time in a packed bed reactor, it is essential first to calculate the active volume inside the reactor. The active volume is the volume available in the reactor for reaction (difference of the volume of the unpacked reactor and the packing material). The concentration or the yield of the desired process are always reported, however, the yield of the side product is generally never reported. It is also desired to measure the concentration of the process stream after the separation stage to check its efficiency [3,21,30,60]. If the desired separation is not achieved then temperature, pressure or scavenger loading should be adjusted to optimize the separation process.

**Table 2:** Basic variables involved for designing multistep flow synthesis and the status of literature on multistep flow synthesis on adapting suitable automation around these variables.

Author/Reference	Reactor						Separator		
			
	Preheating or precooling	T	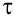	C^a^	P		T	P	C

Hartwig et al. [10]	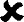	**Y**	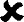 ^b^	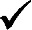	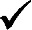		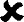	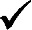	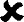
Kupracz and Kirschning [7]	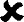	**Y**	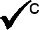	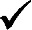	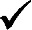		NA	NA	NA
Murray et al. [11]	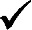	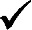	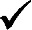	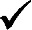	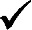		NA	NA	NA
Zhang et al. [14]	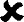	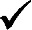	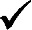	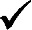	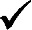		NA	NA	NA
Snead & Jamison [60]	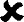	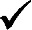	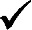	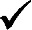	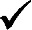		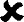	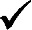	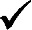
Borukhova et al. [23]	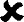	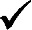	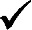	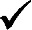	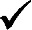		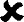	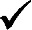	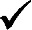
Baxendale et al. [26]	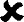	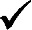	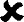 ^b^	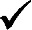	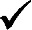		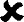	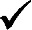	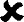
Tsubogo et al. [4]	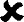	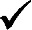	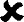	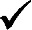	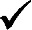		NA	NA	NA
Adamo et al. [3]	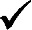	**Y**	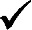	**Y**	**Y**		**Y**	**Y**	**Y**
Poh et al. [84]	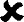	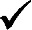	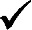	**Y**	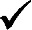		NA	NA	NA
Hartman et al. [30]	**Y**	**Y**	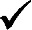	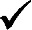	**Y**		**Y**	**Y**	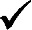
Mascia et al. [24]	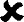	**Y**	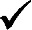	**Y**	**Y**		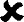	**Y**	**Y**

Symbols used and their meaning: (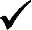 - Parameters are either reported or measured offline, 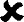 - Parameters are either not reported or not measured and Y - Parameters are measured online or controlled, Superscripts used and their meaning: ^a^Concentration at the reactor outlet or yield of reaction at reactor outlet, ^b^residence time was not reported for the majority of the reactors which belonged to packed bed reactor category and ^c^residence time was reported for the majority of the reactors).

**Challenges in automating special cases:** Each process will have different challenges and it should be addressed separately. Some of the possible challenges are discussed below:

**Handling of solids in flow reactors:** Clogging of solids is a critical problem in a flow reactor. Recently many researchers have investigated clogging phenomena in micro-reactors and capillaries [87–90]. The event of clogging can be monitored by measuring the pressure [15,87]. The pressure will increase as the solids clog the reactor. Ideally one should identify the operating conditions that result in clogging and optimize the reaction such that clogging in the reactor is avoided. However, this may not be always possible and hence it is desired to develop a control strategy that will take appropriate action to address the clogging and to bring the process back to the steady state. For achieving this it is desired to monitor the pressure of the system and to develop a control strategy that will take appropriate action in the event of clogging. Some of the possible strategies can be (1) switch off the valves of the reactants and flush the reactor with an appropriate solvent to remove the clogging or (2) turn on the sonication while the reactants are flowing through the reactor. This will also minimize the power consumption of the sonication system as it will not be switched on continuously. However, before implementing such control strategy, one should have experimental data of pressure vs time to understand the time scales of clogging and the unclogging process. The pressure set point/cut-off value can also be obtained from such data. Alternatively, one has to design the flow reactor taking into account the complex solid–liquid flow for the flow synthesis of Grignard reagent as reported by Wong et al. [91].

**Maintaining temperature below the maximum allowable temperature:** Some reactions like diazotization [92–94], lithiation [7,20], etc. have a maximum allowable temperature as a safety or design criteria. Such a reaction temperature should be monitored along the reactor at strategic locations. The control strategy should take appropriate action if the temperature reaches the maximum allowable temperature to avoid any runaway, decomposition and related hazards.

**Maintaining constant conversion:** It is desired to achieve a fixed conversion at the reactor outlet to maintain a steady state and constant product quality. This is very challenging when the entire system involves a complex network of dependent variables and parameters. This is usually done by controlling the reactor outlet temperature and manipulating the reactor jacket flow rate. The use of inline measuring techniques can help to directly monitor the concentration at the reactor outlet [33]. For some reactions like fast or multiphase reactions, the conversion and selectivity will be more sensitive towards the flow regime, velocity, dispersion, etc. In such cases, the flow rate of reactants should be the manipulating variable and the outlet concentration should be the controlled variable. For the systems where there are restrictions on the reaction temperature, due to safety reasons, the temperature is not the recommended manipulating variable.

## Conclusion

Automation will have a major role to play for converting the laboratory-scale multistep flow synthesis into industrial processes. In fact, when compared to conventional batch processes, these flow processes will be more logical cases for automation. Till date, except a few exceptions, automation in synthesis has always been interpreted as auto-sampling, in-line monitoring, and self-optimization systems. Auto-sampling and in-line monitoring of process variables like temperature, concentration, pressure, pH, etc. will not only improve the productivity of researchers but also improve the reproducibility of the experiments. The possible variation in the results due to minor changes in the set parameters can also be understood more accurately and used for developing a control structure. Reporting these process parameters can increase the quality of the work as well as the reproducibility.

Self-optimizing systems based on machine learning are the new hot topic in flow chemistry literature. While such systems may give the optimum operating conditions, it may not give insights into the progress of the reaction (like concentration and temperature profiles inside the reactor). If these self-optimization systems are also used for generating kinetic data, the kinetic parameters will add more valve to the research and also take the process one step closer to scale-up. Moreover, selecting right optimization algorithm remains critical for minimizing the time and resources used.

In this review, we have critically reviewed some of the important multistep syntheses in the recent past. The results from multistep flow synthesis indicated are promising and automation can bridge the gap between synthetic chemistry and industrial process. It is shown that automation at laboratory scale is very critical from the operational point of view as it will help to reduce the compounding errors in a big way. Automated control systems are not only responsible for executing normal operations like maintaining a process at steady state but also special purpose operations like a start-up, shut-down, change-over, override and emergency situations. Each operation will have a different protocol, and thorough process understanding is essential for developing an appropriate control logic. Dynamic simulations will be useful for studying special purpose operations. We have also proposed the possible automation cum control logic for a few multistep syntheses and critically investigated the individual process.

The analysis of these representative multistep flow syntheses of a few important molecules indicates that the laboratory scale systems and approaches may not be relevant when one would want to extrapolate them for manufacturing. This means that certain critical sections need to be relooked and a process needs to be re-developed so that necessary time scales at each step are optimized. While these steps are always unavoidable, having an automated synthesis platform at laboratory scale will definitely help to know the issues that one would encounter during scale-up or numbering-up. It is certain that, if such excellent case studies use automated platforms, it will definitely help a true ‘lab to market’ translation as reported by Mascia et al. [24] and also appeal the chemical engineers and process engineers to work closely with chemists to make sure that the wonderful creations at laboratory scale are translated into practice.

March of machines in organic synthesis has begun long ago and is becoming more prominent as the curiosity of a creative chemist is trying to explore the molecular signatures across a wide range of time durations right from short-lived femtosecond species to living organisms that have a life cycle of few years to space chemistry that would hide mysteries spanning several light years. To be precise deeper understanding of complex syntheses will demand more creative time [95]. However, the true potential of involving machines on a routine basis for chemical synthesis coupled with in-line automation followed by analysis, decision making for the next experiment and identifying the optimal conditions is very close. This will help to take away routine jobs from the life of creative chemists and make them find time for thinking on complex chemistries. Implementation of automation in laboratory scale synthesis will also generate a huge amount of useful data for the process engineers who will find it relatively easy to transform a new chemistry into a process. The evolution of automation, instrumentation, sensing, machines, wireless control and faster logical platforms that allow hardware to interface with software has reached a stage where chemists can rely on the machine-based synthesis and process engineers can rely on the data that does not include a ‘*possibly ambiguous*’ contribution of human errors. In all, it will save a lot of time to move ahead in further exploration in organic synthesis.
